# Phylogeny and Patterns of Diversity of Goat mtDNA Haplogroup A Revealed by Resequencing Complete Mitogenomes

**DOI:** 10.1371/journal.pone.0095969

**Published:** 2014-04-24

**Authors:** Maria Grazia Doro, Daniela Piras, Giovanni Giuseppe Leoni, Giuseppina Casu, Simona Vaccargiu, Debora Parracciani, Salvatore Naitana, Mario Pirastu, Andrea Novelletto

**Affiliations:** 1 Institute of Population Genetics, National Research Council (CNR), Sassari, Italy; 2 Experimental Zoo-prophylactic Institute of Sardinia, Sassari, Italy; 3 Faculty of Veterinary Sciences, University of Sassari, Sassari, Italy; 4 Genetic Park of Ogliastra, Perdasdefogu, Italy; 5 Department of Biology, University “Tor Vergata”, Rome, Italy; Kunming Institute of Zoology, Chinese Academy of Sciences, China

## Abstract

We sequenced to near completion the entire mtDNA of 28 Sardinian goats, selected to represent the widest possible diversity of the most widespread mitochondrial evolutionary lineage, haplogroup (Hg) A. These specimens were reporters of the diversity in the island but also elsewhere, as inferred from their affiliation to each of 11 clades defined by D-loop variation. Two reference sequences completed the dataset. Overall, 206 variations were found in the full set of 30 sequences, of which 23 were protein-coding non-synonymous single nucleotide substitutions. Many polymorphic sites within Hg A were informative for the reconstruction of its internal phylogeny. Bayesian and network clustering revealed a general similarity over the entire molecule of sequences previously assigned to the same D-loop clade, indicating evolutionarily meaningful lineages. Two major sister groupings emerged within Hg A, which parallel distinct geographical distributions of D-loop clades in extant stocks. The pattern of variation in protein-coding genes revealed an overwhelming role of purifying selection, with the quota of surviving variants approaching neutrality. However, a simple model of relaxation of selection for the bulk of variants here reported should be rejected. Non-synonymous diversity of Hg's A, B and C denoted that a proportion of variants not greater than that allowed in the wild was given the opportunity to spread into domesticated stocks. Our results also confirmed that a remarkable proportion of pre-existing Hg A diversity became incorporated into domestic stocks. Our results confirm clade A11 as a well differentiated and ancient lineage peculiar of Sardinia.

## Introduction

The domestication of the goat (*Capra hircus*) has been one of the major landmarks in the evolution of human modes of subsistence [1], [2], [3]. Besides its valuable dairy products, its portability has made the goat the key element to ensure a stable human settlement in otherwise inhospitable habitats.

The genetic traces of domestication in this species have been first addressed [4] by analyzing the diversity of the mitochondrial D-loop among individuals collected worldwide. These authors showed that the matrilines of the current breeds coalesce to three founders of the so-called haplogroups (Hg's) A, B and C, indicative of a limited number but not a singular drawing of female founders from wild populations. Later, the number of haplogroups grew to 6 (A, B, C, D, F, G [5]), with the newly described Hg F as the most basal one. Each of these found close matches in the alleged ancestor of the domestic goat, the bezoar (*C. aegagrus*) [6].

While Hg A is the most derived lineage, it is also the most widespread and displays a fast and markedly star-shaped radiation, to be interpreted as the result of the success of animals bred in maternal isolation from wild population(s). Given the presence of this Hg in many breeds currently grown worldwide, its phylogeography could potentially be the essential piece of information to reconstruct the spread of maternal lineages. In particular it would help in distinguishing between ancient and recent displacements of stocks. This seems particularly useful as a further characterization of some locales, in addition to the assignment of a reasonable window of time for their introduction in a given area. This additional information can also be an added value whenever such locales are particularly adapted to a specific habitat, are valued as a recreational resource, or when the labeling of the products as “genuinely local” may enhance their economic relevance, thus calling for specific protection and conservation actions [7], [8], [9], [10], [11].

In this context, however, the internal phylogeny of haplogroup A is poorly understood, mainly because of the hypermutability of the D-loop of mtDNA, which causes many positions to display the same nucleotide by the repeated occurrence of the same mutational event (identity by state) rather than by descent. The net result is a set of inextricable reticulations when one attempts to reconstruct internal, evolutionarily meaningful lineages. As an intermediate step forward, we [12] took advantage of the demography underlying a large proportion of the population of the Italian island of Sardinia. By sampling a large number of animals we identified, within Hg A, subgroups of D-loop sequences related in a star-like fashion, reminiscent of common descent from a limited number of founders. We then defined 11 “ad interim” clades of Hg A, and listed a reduced number of mutations, among those occurring in the D-loop, that can distinguish them. Their frequencies ranged 1.8–21% among >1,500 Hg A individuals. By comparing our results with those of other screenings [5], [13] we also realized that, with the exception of two clades (A-9 and A-11), the same groupings accounted for a large proportion of the stocks grown elsewhere. While this result can be explained by a relatively recent (historical) import of matrilines (possibly conveyed by different breeds) in Sardinia, the presence of peculiar lineages might be due to a much earlier introduction. These lineages might have been rare even in their homeland at the time of introduction in Sardinia and now lost outside the Island, or they might be the result of new mutations that occurred and were selected *in loco.*

In summary, Sardinia recapitulates the D-loop phylogeny of a non trivial portion of Hg A and thus offers the opportunity of working out a clearer picture of divergence within the entire haplogroup. We then selected animals carrier of each of the 11 Hg A clades, plus an Hg C outgroup, and sequenced their mtDNA to near completion. In this way we could test our initial hypothesis that D-loop clades are predictors of evolutionarily meaningful lineages. This question is of general interest as far as the D-loop is the DNA region most commonly addressed to initially approach a number of issues of species' biology (see e.g. Chapter 21 in ref. [14]). Also, finding a large number of variants along the entire mtDNA sequence can identify major evolutionary forces affecting livestock populations after domestication. This includes a better understanding of the selective regime(s) that impacted the mitochondrial genome. We then asked: i) whether our set of variants fitted a model of relaxed selection allowing for otherwise mildly deleterious alleles, as hypothesized for other species [15], [16], and ii) whether a reduced number of mutational changes or assemblages of mutational changes was subjected to positive selection, possibly due to their potential physiological relevance.

In the context of the resulting phylogenetic reconstruction, the clade A-11 of Hg A emerges as an ancient lineage, currently peculiar of Sardinia, characterized by many non-synonymous substitutions.

## Results

The sequence coverage attained in the 28 individuals selected for complete mtDNA resequencing (Table S1 in File S1) is summarized in Figure S1, with colors indicating Hg A clade affiliation. With the exception of a single clade A8 individual (id. 304) sequence coverage was always above 80%. For all clades at least one individual was covered at >89%. For the single Hg C individual only a 336 bp portion of the sequence was missing (98% coverage).

Only two regions remained poorly covered in several individuals, i.e. those at the beginning of amplicon 3 and at the end of amplicon 31. In the first case the presence of a C_10_ stretch often caused slippage that generated overlapping shifted profiles further downstream (this problem was not observed in carriers of an interrupting T at pos. 1114). In the second case the complete coverage of the large amplicon 31 was attempted by sequencing in both directions, but profiles obtained with the reverse primer turned out to be often short or of poor quality and then discarded.

As one of our aims was to explore the phylogenetic reliability of the relationships between Hg A clades defined by D-loop sequences, our entire analysis took into account only variations in the region spanning reference positions 1–15,430 (i.e. outside the D-loop). In addition to our Hg C individual (id. 985), all our analyses included sequences GU068049 (representative of Hg A from Mongolia) and GU295658 (representative of Hg B from South East Asia), thus reaching a total of 30 sequences hereafter referred to as the “full set”.

Overall, 206 variations were found in the full set of 30 sequences (Table S2 in File S1). Of these, 113 were contributed by the Hg C and B sequences, whereas 93 were found among Hg A sequences. This confirmed Hg's C and B as strongly divergent lineages, but the number of residual variable positions denoted that many polymorphic sites within Hg A could be informative for the reconstruction of its internal phylogeny. In fact, 59 of the 93 variations were singletons in Hg A individuals and the remaining 34 were shared by two or more individuals. Eleven variable positions were private for the reference sequence GU068049, 7 of which residing in regions covered in ref. [17] and also reported therein.

Among single nucleotide substitutions 193 and 8 were transitions and transversions, respectively. Among transitions T/C changes outnumbered A/G changes (146 vs. 47). As to indels, two consecutive thymine residues reported for the reference sequence GU068049, were not found in any other sequence and were considered as two additional adjacent private single nucleotide indels (positions 177, 178). Two variations consisted in length changes in low complexity A_8_ (pos. 11560-7) and C_10_ (pos. 1111-20) stretches. In particular this latter segment was C_11_ in Hg B but shortened and affected by other substitutions in Hg C. Finally, an A insertion was found in a single individual, between positions 1238 and 1239.

### Reconstructing Hg A phylogeny

We used two independent methods to obtain a reconstruction of affinities between mtDNA sequences. The first method was a Bayesian tree reconstruction. The results are displayed in Figure S2. The relative order of branching of Hg's C and B replicates that of refs. [4], [5], [17] with highly supported nodes, and identifies Hg C as the most basal lineage. The next node (support 100%) defines the radiation of all Hg A lineages and separates the reference sequence GU068049 from the rest of Hg A sequences. A series of closely spaced nodes then separates small groupings. Each of these is formed by sequences clustered together with high support and previously assigned to the same D-loop clade. The only exceptions are represented by an A5 sequence (id. 1495) clustering with two A7 sequences, another A5 (id. 331) defining a single branch outside the clade of the remaining A5 sequences, and the two A8 sequences (id's 830 and 304) which did not cluster together. Of the three sequences not previously assigned to any of the D-loop clades, two (id's 1332 and 480) were highly similar in the D-loop and clustered together in the tree, while the third (id. 692) was divergent in the D-loop and clustered with A9 in the tree.

In summary, this clustering scheme denoted a general similarity over the entire molecule of sequences previously assigned to the same D-loop clade. As to the relationships among clades, these remained less strongly supported and partly contradicted our previous reconstruction (see Fig. 2 in [12]), most likely because of the star-like and rapid divergence between them, and the presence of nucleotide states generated by recurrent mutational events (see below). The 27 Sardinian Hg A sequences here examined clustered into two sister groupings downstream to a poorly (0.21) supported node. However we noticed two features of this partition that may strengthen its significance. First, it separated the sequences in the same manner as a major hiatus in the D-loop network (clades A1 to A6 plus A9 in Fig. S2, bottom vs. A7 to A11 in Fig. S2, top) and second, it had a clear geographic correlate (Fig. S2). In particular, the sequences of the first group turned out to be associated with D-loop clades represented mainly in Europe when inspecting three datasets [5], [13], [18], with only a minority of observations from Asia. Conversely, the sequences of the second group appeared to be associated with three clades (A7, A8 and A10) abundant throughout Asia. Sequences corresponding to D-loop clade A11 were assigned to this latter group (Fig. S2, top). Indeed a single instance of the haplotypic arrangement of D-loop variants characteristic of clade A11 was represented among the 2488 individuals in the datasets of refs. [5], [13], and it was sampled in Italy. Conversely, its common presence in Sardinia was confirmed in an independent study [18].

**Figure 1:**
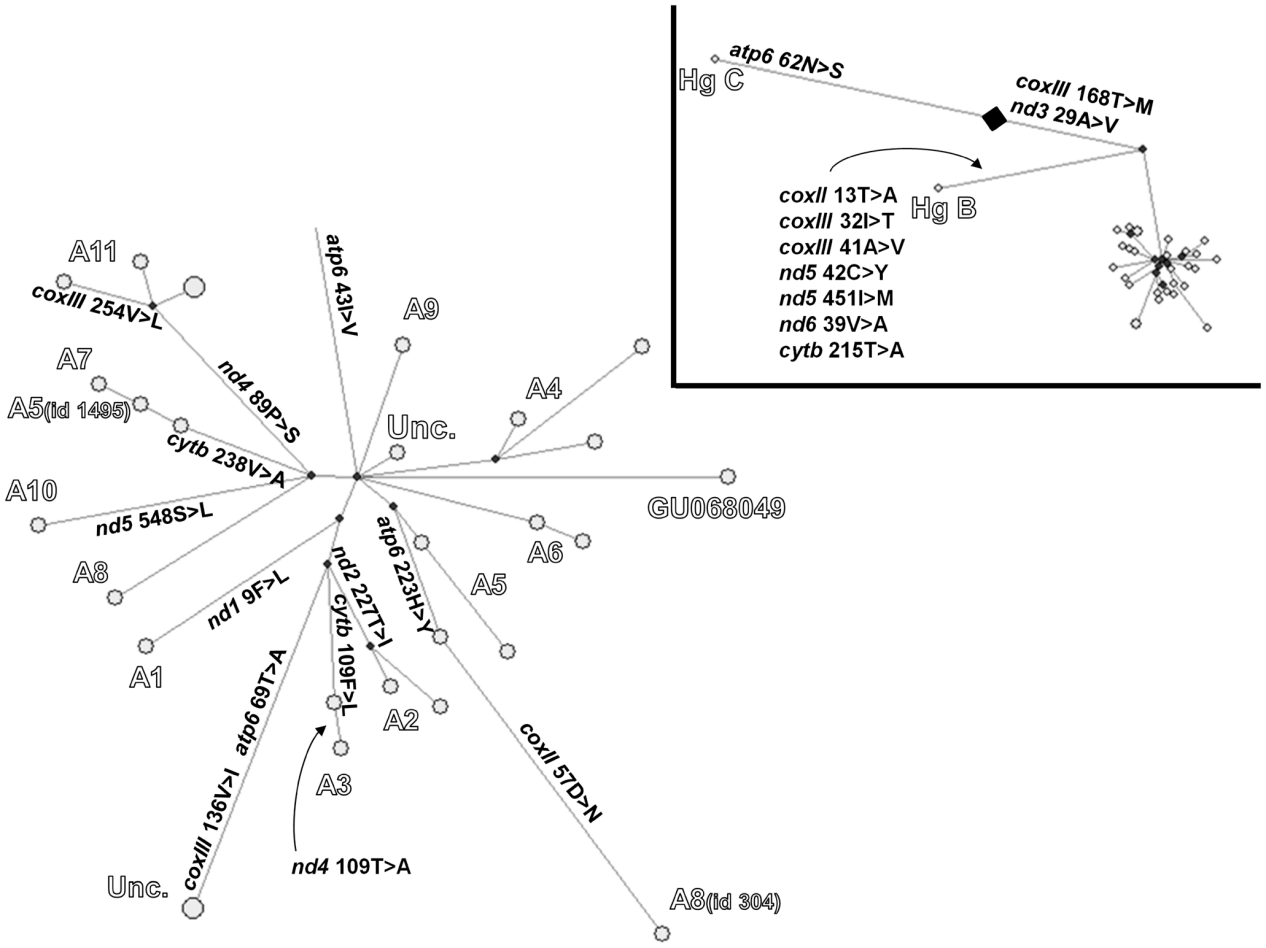
Median joining network of 30 sequences showing the occurrence of 23 non-synonymous substitutions. Each circle represents a sequence. Branch length is proportional to the number of mutations. Note that two A11 and two uncharacterized sequences are lumped into single (larger) nodes (see Materials and Methods). The haplogroup or D-loop clade (for Hg A) affiliation of clusters of sequences is shown in large empty characters. Aminoacid replacements are shown beside each branch in their polarized form. The Hg A portion of the network is magnified while the original network is in the inset. A large lozenge indicates the likely position of the root and was added in the drawing to partition three variants peculiar of Hg C. Their polarization was obtained by comparison with *C. ibex*.

We also constructed a network representing the arrangement of allele states among our sequences (Fig. 1). Since we excluded some positions, the topology of the network turned out to be simpler than the Bayesian tree. Nevertheless, the branching order of Hg's C, B and A was replicated (Fig. 1, inset), with a large multifurcation of all Hg A sequences. The network also replicated the clustering of sequences according to their D-loop clade affiliation already evident in the Bayesian tree, with a single exception represented by id. 304 (clade A8) which now appeared at the tip of a long branch distal to an A5 sequence (Fig. 1, bottom right). Interestingly, one short branch of the multifurcation led to a node basal to all sequences of clades A8, A7, A10 and A11 (plus A5, id 1495), i.e. one of the major groupings in the Bayesian tree. The network construction identified 7 positions with recurrent mutational events (Table S2 in File S1).

### Exploring possible selection regimes

We searched for genetic traces left by selection regimes in the whole set of lineages here considered by analyzing separately protein-coding and RNA-coding genes.

**Figure 2:**
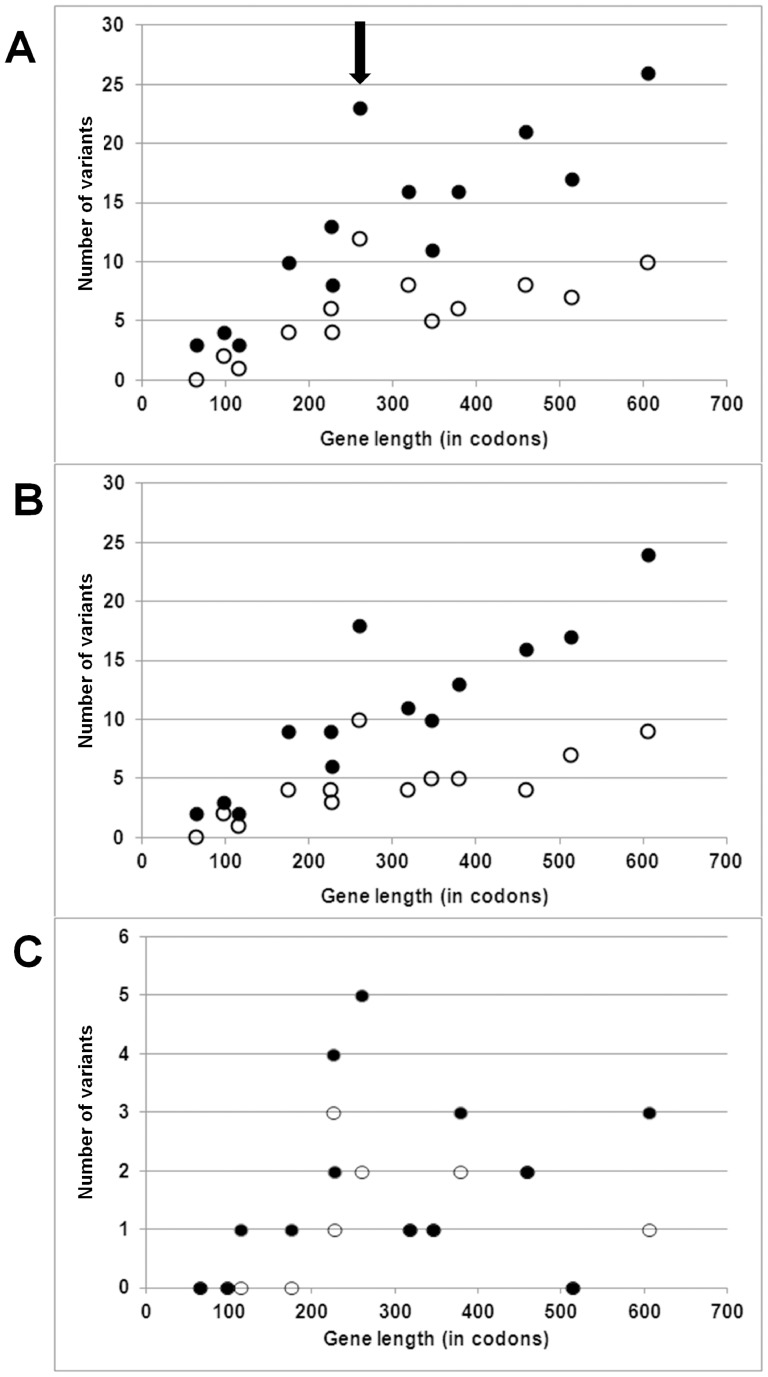
Scatterplot of number of variants recorded as a function of length of each of the 13 protein-coding mtDNA genes. Filled dots: variants scored among all 30 sequences considered. Empty dots: variants scored among the 28 Hg A sequences. Panel A: all positions. Panel B: only 3^rd^ codon positions. Panel C: only non-synonymous variants (note that the three black dots at 0 variants overlap and obscure empty dots). *coxIII* is indicated by an arrow.

As to protein-coding genes, we argued that if variants in our set were largely neutral, they should be found in all genes and in numbers proportional to the targetable positions in each gene. This expectation was verified, with a linear and highly correlated increase of variants as a function of gene length, both including (R^2^ = 0.71; p = 3×10^−4^) and excluding (R^2^ = 0.52; p = 5×10^−3^) the large number of variants contributed by the Hg B and C sequences (Fig. 2A). This observation was in large part attributable to variants in third codon positions, which accumulated linearly (Fig. 2B) with gene length in both the full set of 30 sequences (R^2^ = 0.81; p = 3×10^−5^) as well as in the 28 Hg A sequences (R^2^ = 0.51; p = 6×10^−3^). In both cases *coxIII* displayed an unusual accumulation of variants.

The linearity between the number of variants and gene length was somewhat disrupted for non-synonymous variants (Fig. 2C), with low correlations for both the full set of 30 sequences (R^2^ = 0.06, p = 0.41) and the 28 Hg A sequences (R^2^ = 0.08, p = 0.33). The highest absolute numbers of non-synonymous variants were in *coxIII* and *atp6* (total length  = 280 and 226 codons, respectively) whereas the relatively long *coxI* (514 codons) harboured no variants.

In order to distinguish whether this result was due to stochastic variations in the small numbers of non-synonymous variants or differential selection across genes, we analyzed the data in a McDonald-Kreitman framework [19]. For each gene we then compared non-synonymous and synonymous variations found among Hg's A, B and C (polymorphic) or only in Hg A, with those recorded in the divergence with *C. ibex*. The results are shown in Table S3 in File S1. For none of the genes the McDonald-Kreitman test turned out to be significant, either considering the three major haplogroups or only Hg A.

Thus, in summary, the absolute number of non-synonymous as compared to synonymous variants was in line with a generalized purifying selective regime on the former ones, possibly with variable intensities across genes, as exemplified by the accumulation above average in *coxIII* (21.7% of non-synonymous variants). However, when compared with the wild outgroup, the proportions of the two types of variants showed that, quantitatively, the intensity of purification remained unaltered under domestication.

The above results could not rule out that only a small subset of non-synonymous variants were impacted by the breeding practices. In fact, under domestication, selection on these variants may have become less stringent or null (see e.g. [15]), possibly as an effect of breeding practices that favored the survival of females and drove the demography of matrilines [20]. A second hypothesis is that diversifying selection operated by breeders maintained a repertoire of valuable phenotypes influenced by some of the non synonymous variants.

We analyzed the occurrence of non-synonymous variants in the phylogeny, to rule out possible cumulative effects of variants with subtle phenotypic effects. Of the 23 non-synonymous substitutions observed in the full set of 30 sequences (Table 1), 3 were peculiar of Hg C (Fig. 1, inset). For one of these Hg C carried the derived aminoacid state (*atp6* 62S), whereas for the remaining two Hg C carried the ancestral state (*coxIII* 168T and *nd3* 29A) and the derived alleles were shared by Hg's B and A (*coxIII* 168M and *nd3* 29V). The excess of 7 non-synonymous vs. 20 synonymous changes on the branch leading to Hg B (matching the increased ω ratio reported in this Hg reported in [17]), was not significant (p = 0.06; Fisher exact test) as compared to the rest of variants in protein-coding genes.

**Table 1:** Comparative analysis of the 23 non-synonymous substitutions in protein-coding genes inferred in the full set of 30 sequences.

Position	Gene	Allelic variants	aa. replacement	Homologous aa. in rCRS	Variants in MITOMAP^1^	Occurrences in GB	MutPred pathogenicity score in human^2^	mtDNA selection score in human^2^
2767	*nd1*	T/C	F9L	L	*L-L*	23	0.625	0,768
4587	*nd2*	T/C	T227I	T	T-A	1	0.586	0.661
7053	*coxII*	A/G	T13A	T	*T-T*	122	0.378	0.297
7185	*coxII*	A/G	D57N	D	**D-N**, D-A, D-G, D-D	10, 1, 1, 3	0.499	0.473
8056	*atp6*	A/G	V43I	I	**I-V**, I-T, I-I	9, 5, 1127	0.325	0.242
8114	*atp6*	A/G	N62S	N	**N-S**	6	0.526	0.525
8134	*atp6*	A/G	T69A	S	*T-T*	7	n.a.	n.a.
8596	*atp6*	T/C	H223Y	H	H-R	3	0.656	0.865
8704	*coxIII*	T/C	I32T	A	A-V, A-A	8, 26	n.a.	n.a.
8731	*coxIII*	T/C	A41V	T	T-A, T-M, T-T	7, 1, 33	n.a.	n.a.
9015	*coxIII*	A/G	V136I	V	*V-V*	8	n.a.^3^	n.a.^3^
9112	*coxIII*	T/C	M168T	L	L-L, L-S	17, 1	n.a.	n.a.
9369	*coxIII*	C/G	V254L	V	V-I, V-V	117, 9	0.471	0.425
9548	*nd3*	T/C	V29A	G	G-S	66	n.a.	n.a.
10433	*nd4*	T/C	P89S	L	L-P, L-L	64, 4	n.a.	n.a.
10493	*nd4*	A/G	T109A	T	T-P, **T-A**, T-T	n.a., 109, 6	0.482	0.443
11872	*nd5*	A/G	C42Y	S	*S-S*	3	n.a.	n.a.
13100	*nd5*	A/T	I451M	L	*L-L*	5	n.a.	n.a.
13390	*nd5*	T/C	S548L	L	*L-L*	62	n.a.^3^	n.a.^3^
13964	*nd6*	A/G	V39A	V38	V-L, V-I	2, 6	0.544	0.562
14479	*cytb*	A/T	F109L	Y	Y-N, Y-H, Y-C	n.a., 33, 1	n.a.	n.a.
14795	*cytb*	A/G	T215A	S	*S-S*	19	n.a.	n.a.
14865	*cytb*	T/C	V238A	S	S-P, S-F, S-S	28, 7, 8	n.a	n.a.

1 Non synonymous variants are bolded. For positions in which only synonymous variants are known italics is used;

2 from ref. [22];

3 same aminoacid encoded by a different codon in rCRS and goat;

n.a.  =  not applicable.

One substitution (*atp6* 43I>V) was shared by all Hg A sequences but not Hg's B and C and was then assigned to the branch basal to Hg A, with the derived state in this Hg in agreement with previous results [17].

Whithin Hg A, a non-synonymous substitution was found on each of the branches basal to sequences affiliated with D-loop clades A1, A2, A3, A7, A10 and A11, while two were on the branch leading to the highly similar uncharacterized sequences id. 1332 and 480. This co-occurrence of aminoacid substitutions in *atp6* and *coxIII* represents a non-significant excess (p = .38, Fisher exact test) as compared to 4 and 5 substitutions in each gene, respectively, over the 37 branches of the network. Additional private non-synonymous substitutions were found for sequences affiliated with clades A8 (id. 304), A3, A5 and A11. Finally, an additional non-synonymous substitution (*nd5* 586L>Q) was found in one of the 5 supplementary clade A11 individuals sequenced in amplicon 28. In summary, a non-synonymous substitution was peculiar of all Hg A sequences and additional 12 affected 18 of the 28 Hg A sequences here considered, ruling out the selection of functionally highly diversified lineages.

We also reasoned that the two selective regimes outlined above lead to different expectations as to the composition of the repertoire of non-synonymous variants, with an enrichment of mildly deleterious variants under relaxed selection vs. an absence of detrimental variants under diversifying selection. In order to gain insights on the features of our array of non-synonymous variants, we explored the homologous positions in the largest collection of mtDNA mutations recorded so far for a single species, i.e. the MITOMAP database of *Homo sapiens*. For each DNA position this database reports the observed variant(s), the corresponding aminoacid change, the number of occurrences of each variant in GenBank (GB, to be used as a rough estimate of its degree of polymorphism) and the phenotypic consequences of each mutation, if available. The results are reported in columns 5–7 of Table 1. In nearly half of cases (12/23) one of the aminoacids observed among our variants was conserved in the orthologous position of the human reference sequence (rCRS). Four of our aminoacid substitutions were replicated exactly in humans. Three of these had 10, 9 and 6 records in GB, respectively, i.e. values above the 60th percentile of frequencies among the 7932 variants. Interestingly the fourth (*nd4* T109A) was initially associated with the MELAS syndrome. However it was also found at high frequency in normal controls [21]. Its causative role in the disease is now questioned (http://omim.org/entry/516003). Among our animals, this variation was found in only one of the two A3 individuals, and its frequency in the Sardinian stock at large remains to be determined. Overall, in the set of 13 protein-coding genes, the ratio of non-synonymous to synonymous variants recorded in MITOMAP approached that observed here only when rare variants were excluded (considering only variations found >40 times in GenBank, Fig. S3).

In 8 of the 23 human orthologous positions (34.7%) only synonymous variants were recorded, a finding potentially denoting that aminoacid replacements at these positions are not tolerated. Indeed this observation does not deviate from expectations, as far as the proportions of positions displaying only synonymous variations across the human orthologues of the 10 genes listed in Table 1 range between 21.9% in *atp6* and 63.7% in *nd4*.

Finally, we considered the indicators of possible pathogenicity computed in ref. [22] for all possible aminoacid substitutions in the human rCRS. The 10 substitutions that would replicate those observed in goats all display MutPred pathogenicity scores below the mode of the overall distribution and selection scores lower than 1, indicative of effects much milder than mutations recognized to be pathogenic. Even the *nd4* T109A, the sole substitution which appears in the list of 75 pathogenic mutations, has scores as low as 0.482 and 0.443 (Table 1).

When feasible, we repeated the same type of comparative analyses for variants in RNA-coding genes (Table 2). For variants in rRNA genes, 8 high-confidence orthologous positions displayed high frequency variants in human, too. The fact that for 5 positions there are no variants recorded in MITOMAP is within expectations, as far as this database contains only 271 and 438 variable positions for the 945 and 1569 bp of the 12S and 16S rRNA, respectively.

**Table 2:** Comparative analysis of the substitutions in RNA-coding genes inferred in the full set of 30 sequences.

Position	Gene	Allelic variants	Homologous pos. in rCRS	Variants in MITOMAP	Occurrences in GB
165	12S-rRNA	T/C	743C	not present	
177	12S-rRNA	T/del	amb. align.^1^		
178	12S-rRNA	T/del	amb. align.^1^		
180	12S-rRNA	G/T	amb. align.^1^		
181	12S-rRNA	A/T	amb. align.^1^		
196	12S-rRNA	T/C	773T	T-C	1
205	12S-rRNA	T/C	782A	not present	
213	12S-rRNA	T/C	790A	not present	
605	12S-rRNA	T/C	1187T	T-C	18
944	12S-rRNA	T/C	1520T	T-C	13
1026	12S-rRNA	T/C	1601C	C-T	2
1114	16S-rRNA	T/C	amb. align.^1^		
1111-20	16S-rRNA	C_10_/C_11_			
1192	16S-rRNA	T/C	1764C	C-T	1
1238/39	16S-rRNA	A/del	amb. align.^1^		
1369	16S-rRNA	T/C	amb. align.^1^		
1671	16S-rRNA	A/G	2241A	not present	
1850	16S-rRNA	T/C	2417C	C-G	4
2120	16S-rRNA	T/C	2686G	not present	
2190	16S-rRNA	A/G	2756C	C-A, C-T	1, 5
2208	16S-rRNA	T/C	2774C	not present	
2619	16S-rRNA	T/C	3184C	C-T	1
3771	tRNA-Gln	T/C	4335C	C-T	9
3892	tRNA-Met	T/C	4454T	T-A, T-C	90, 80
6894	tRNA-Ser (UCN)	T/C	7468C	C-T	5
7723	tRNA-Lys	A/G	8315A	A-G	1
9827	tRNA-Arg	T/C	10421C	C-T	1
9860	tRNA-Arg	T/C	10454T	T-C	59
9863	tRNA-Arg	A/G	amb. align.^1^		
11560-7	tRNA-His	A_8_/A_9_	amb. align.^1^		
11569	tRNA-His	A/G	12160A	not present	
11624	tRNA-Ser (AGY)	T/C	12214C	not present	
11691	tRNA-Leu(CUN)	A/G	12279A	A-T	1
11702	tRNA-Leu(CUN)	A/G	12290A	A-G	2

1 Ambiguous alignment.

Also the orthologues of variants residing in tRNA genes appeared to be highly polymorphic in human, with the replication of the alternative alleles in 7 out of 8 cases.

## Discussion

In this work we proceeded a step forward in the description of the phyletic relationships between lineages belonging to a major component of the goat mitochondrial diversity, Hg A. We obtained this by leveraging the diversity represented in a single Italian region, where the goat is highly valued and its diversity preserved.

We restricted our analysis to a portion of the molecule less prone to recurrent mutations than the highly variable D-loop (115 variable positions in 1213 bp across our 30 sequences). Nevertheless we showed that clusters of sequences defined by a selected subset of D-loop mutations [12] do match clusters defined by variants in the rest of the molecule. The topology connecting these clusters needs additional support, which can be obtained by completion of the typing of missing positions in our set. In particular, dubious assignments involve the lineages associated with the D-loop clades A5 and A8, which probably still contain a large amount of internal diversity. As to A9 we [12] already warned that this has to be considered mainly a paragroup, as it is defined only by ancestral alleles in the D-loop.

Also, the inclusion of high quality complete Hg A mitogenomes from outside Sardinia in future analyses is imperative. In fact, only three (7214T/C, 9221T/C and 14518A/G) of the variable positions here reported were also present among the 62 positions variable in Hg A animals sampled mainly in eastern and south-eastern Asia [17], and allele states at two of these were probably homoplasic. Interestingly, also in this latter study the peculiarity of the reference sequence GU068049 (from the Inner Mongolian breed) emerged, with four private variants and three variants shared with a single individual (sample ID G1307). These findings qualify the trees of the two studies as complementary subsections of the entire Hg A phylogeny, but also predict that northern Asia may represent a further and poorly explored reservoir of goat mitochondrial diversity. Only when a robust topology will be attained it will be possible to reconsider the diversity of the D-loop and to enumerate the recurrent events within it.

With the above caveats in mind, a major branching among the Hg A sequences here reported separates lineages currently found mostly in western Eurasia from lineages common in the eastern Old World. Phylogeographic considerations in this species are weakened by its high portability by both land and sea since several millennia [23], which may have led to the seeding of widely distant regions of the world in a manner disconnected from phyletic affinities (see e.g. [24]). Despite this, the present study captured a partition probably reflecting an ancient split in the spread of stocks carrying Hg A from its likely homeland in Eastern Anatolia [6]. This hypothesis may serve to orient the search of markers in archeological material that documents that process. As far as genetics is concerned, D-loop clade assignment was feasible in at least 89% of 29 Hg A bezoars [6] and identified 17 and 8 carriers of clades A8 and A5, respectively (Table 1), corresponding to each of the major Hg A tree branches. While the former animals were largely sampled in Iran (14 vs. 3 in Turkey) the latter ones were from Iran and Turkey in equal proportions, replicating to some extent the same east-west imbalance in the distribution of domestic goat lineages.

Our results also confirm the view that a remarkable proportion of pre-existing Hg A diversity became incorporated into domestic stocks. A date between 201 and 281 thousand years ago was proposed [4] for the coalescent of Hg's C, B and A. In our Bayesian tree Hg A occupies approximately 15% of the total tree height, corresponding to a date far predating the beginning of domestication. Using the average rate proposed [25] for 3^rd^ codon positions in wild Caprinae results in a similar estimate (File S1). This discrepancy between the age of the molecules and that of the domestic stocks testifies of a process of domestication not accompanied by strong bottlenecks and loss of matrilinear diversity, extending previous results obtained in the goat [6], [17]. Speculations on which mechanism(s) produced this outcome include multiple drawings also for Hg A, from populations less differentiated that those harboring Hg's C and B. This could have occurred during a long period preceding full-blown domestication, in which semi-wild populations were simply managed [3], [6], [26], [27], without interrupting gene flow with wild relatives. It is worth noting that this represents one of the extremes of a full spectrum of possibilities. At the opposite end lies the taurine cattle (*Bos taurus*) for which an extremely narrow bottleneck was inferred based on mtDNA data [10].

Overall, the pattern of variation in protein-coding genes revealed and confirmed an overwhelming role of purifying selection [15], [17], with the quota of surviving variants approaching neutrality. The features of the set of 23 non-synonymous variants did not provide any evidence for an enrichment in deleterious aminoacid replacements which have approached neutrality due to relaxed selection. Conversely, they replicated a group of variants which have been found to reach polymorphic frequencies in humans [22]. Interestingly, a large proportion of non-synonymous substitutions were on branches with two or more sequences downstream (internal branches), an additional evidence of their neutral or quasi-neutral behaviour. The overall proportions of synonymous variants here reported for Hg's A, B, C or Hg A only (0.865 and 0.838, respectively; Table S3 in File S1, bottom) closely approach the asymptotic values reported for a number of mammalian taxa in ref. [28]. These estimate the constant corresponding to the observed proportion of synonymous mutations after the effect of purifying selection has become negligible. In our dataset the applicability of formal tests on the drasticity of aminaocid replacements (e.g. as implemented in TreeSAAP, [29]) was limited by the reduced number of variants in each gene.

A similar argument applies to RNA-coding genes, as well. Here we found a number of variants per kilobase (11.5, 6.9 and 8.0 for 12S rRNA, 16S rRNA and tRNA's, respectively) even lower than in protein-coding genes (15.0), in line with the strong purifying selection inferred in other species [15]. However, the few observed variants have polymorphic homologous variants in humans.

In summary, a simple model of relaxation of selection for the bulk of variants here reported should be rejected. Rather, our analysis of non-synonymous diversity (which considered intra- vs. inter-species instead of intra- vs. inter-haplogroup [17] comparisons) revealed that a proportion of variants not greater that that allowed in the wild was given the opportunity to spread into domesticated stocks. This also excludes a regime of generalized diversifying selection. Mitochondrial phenotypes have been claimed to play a role in a number of life-history traits [30], [31]. A reduction of metabolic efficiency was hypothesized to be favourable in a carnivore, the dog [16], but this seems hardly transferable to a ruminant, whose valued products are milk, fat and lean body mass and a high reproductive rate. Of main interest here are instead temperature and starvation resistance, in light of the new environmental conditions encountered by the goats along with their breeders' migrations. An approach to reveal possible positive selection on individual variants will be to measure diversity indexes in several sequences from the same clade. This has not been feasible here, due to the highly biased criterion for inclusion of individuals in the study.

Our results now confirmed that clade A11, (previously recognized as peculiar of Sardinia [12], [18] where it reaches frequencies as high as 8.6%), indeed identifies a lineage that is well differentiated along the entire mtDNA molecule and is not simply a derivative of one of the lineages confined to Europe. Based on the current distribution of mtDNA lineages mostly related to it, the possible geographical range for the origins of clade A11 sequences is much wider than Europe alone. Moreover, based on the number of mutations distinguishing it, its antiquity is comparable to that of much more common lineages currently spread from Europe to East Asia. This suggests that goats carriers of this lineage were relocated into the Island from populations that are now lost or not yet explored for mtDNA diversity.

## Materials and Methods

### The individuals

All activities carried out by the Regional Association of Sardinian Farmers (ARAS) officers, part of which related to this study, followed ethical guidelines for care and use of agricultural animals for research (EC Directive 86/609/EEC). For this study specific approval by a review board was not necessary, as none of the procedures used here met the criteria to define them experiments as defined in Article 2 of the cited Directive. All animals of the present study belong to the series previously studied [12]. They were not re-sampled and the new data were generated on previously collected DNA, without any additional burden to the animal.

Twenty-eight individuals were selected to represent all the clades reported in [12], based on D-loop diversity. In order to represent also the within-clade diversity, individuals belonging to the same clade were included only if they differed at some positions in the D-loop. Three individuals were included specifically to represent the unclassified D-loop sequences.

Additional criteria for inclusion in the above group of 28 individuals were: a general phenotype fitting that of the “Sarda” breed [32], and a geographical origin preferentially in the Ogliastra sub-region of Sardinia (see Fig. 1 in [12]).

In order to confirm/dismiss a subset of mutations, additional 38 individuals belonging to D-loop clades A2, A4, A5, A7, A8 and A11 were considered and sequenced for amplicons 10, 22 and 28 (see below). This group, too, was selected to display the “Sarda” phenotype, but the geographical criterion was relaxed and representatives of other sub-regions were included. The essential data are reported in Table S1 in File S1.

### Amplification and sequencing

The entire mtDNA was subdivided into 33 partially overlapping amplicons (Fig. S1 and Table S4 in File S1). Amplifications were performed in a total volume of 15 ul in the presence of 1x reaction buffer, 1.5 mM MgCl2, 7.5 pmoles of each primer, 200 uM dNTPs, 1 ng of genomic DNA and 0.5 U Taq polymerase. Thermal profiles consisted of 5 min. at 96°C, 35 cycles of 30 sec. at 95°C, 30 sec. at the annealing temperatures reported in Table S4 in File S1and 1 min. at 72°C, followed by a final extension of 10 min. at 72°C. Five ul of the PCR product were then incubated 15 min. at 37°C with exosap, followed by 15 min. at 80°C. Sequencing reactions were performed with the ABI PRISM BigDye Terminator kit (v1.1, Applied Biosystems) in the presence of 1.7 pmoles of primer F (and R for amplicon 31) for 10 min. at 96°C, 25 cycles of 5 sec. at 55°C and 2 min. at 60°C. Products were separated in a PRISM ABI 3130xL Genetic Analyzer. Low quality sequence profiles were discarded upon visual inspection. High quality profiles were aligned with the Lasergene SeqMan software together with sequences representing haplogroups A to D (accession nos. GU068049, GU229278, GU295658, GU229279, GU229280, GU229281).

Throughout the text position numbering refers to sequence GU068049. All sequences are deposited in GenBank with Acc. n. KJ192209-KJ192236.

Two individuals (id. 1237 and 1300) displayed ambiguous peaks at pos. 4587 (Fig. S4). As an unambiguous T was observed in 6 additional individuals it was considered a real variant. However the T/C ambiguity (Y) was retained in all analyses involving id. 1237 and 1300.

### Data analysis

A Bayesian tree reconstruction was performed with BEAST [33]. We used all the 28 nearly complete sequences between positions 1 and 15,430, i.e. outside the D-loop, retaining all uncertainties present in the original data. In this analysis we included the reference sequences GU068049 and GU295658, obtained from an Hg A and an Hg B individual, respectively, but excluded the other sequences reported in ref. [34] to be unexpectedly divergent or chimeric (final whole set of 30 sequences). A number of preliminary runs were used to set the priors, a suitable starting tree and to explore the possibility of estimating absolute dates for the tree nodes (see File S1). In the final analysis we used a substitution rate of 1 to obtain branch lengths in mutational units. We then used a GTR+Γ+i substitution model with strict clock and constant population size for a 10-million step run, sampled every 10,000 steps. After discarding a 50% burn-in, the results were inspected with Tracer, condensed with TreeAnnotator and visualized with TreeFIG as recommended in the user manual.

In order to obtain an independent interpretation of the phylogentic relationships between sequences and to precisely locate mutations on branches, we constructed a median joining network [35]. In order to avoid a large number of unsupported reticulations, in this analysis we removed all positions not typed in 9 or more of the 28 individuals, plus 6 problematic positions (177, 178, 605, 4606, 9600, 11567) in which the inferred number of state changes did not fit that immediately deduced upon visual inspection (due to the additional presence of missing data in some of the sequences). The statistics file produced by the program was used to identify positions undergoing multiple mutational changes along the network. The assignment of mutations to each branch is reported in Table S2 in File S1, with branches numbered as in Fig. S5.

The assignment of D-loop sequences from external datasets to the same clades here used was based on the presence of haplotypic arrangements of variants described in Table 2 of [12]. For each of the cited datasets, the full set of sequences was aligned with a non-redundant subset of those in ref. [12], and allelic states compared after renumbering.

Synonymous and non-synonymous substitutions were identified by translating the DNA sequences in MEGA [36]. The counts of non-synonymous and synonymous variants used in the McDonald-Kreitman tests [19] were obtained by aligning the DNA sequences of the 13 protein-coding genes in GU068049 with the orthologues of *Capra ibex* isolate G1253 (Acc. n. AB743814.1 to AB743826.1). From the raw counts we excluded all positions for which the difference between C. *ibex* and *C. hircus* GU068049 was attributable to the presence of a derived polymorphic allele in the latter, thus leaving only variants fixed before the divergence of Hg's C, B and A.

The complete list of mtDNA variants recorded at MITOMAP (http://www.mitomap.org/MITOMAP) as of Aug. 30, 2013 was downloaded and analyzed in a spreadsheet. This contains data for 7932 DNA variants obtained from 18363 GenBank (GB) sequences with size greater than 14 kb. To ensure that orthologous positions at the aminoacid level were compared, alignments of goat and human protein sequences were obtained with COBALT (http://www.ncbi.nlm.nih.gov/tools/cobalt/), using the protein id's in GU68049 and in the human revised Cambridge Reference Sequence (rCRS, Acc. n. NC_012920). For RNA-coding genes, a multiple alignment of GU068049, GU295658 and the rCRS sequences was generated with Clustal [37], with default settings. For each position variant in the goat, a surrounding range of 40 bp was re-aligned and visually inspected to exclude instances with multiple alignment solutions.

The list of all possible aminoacid substitutions in the rCRS [22] was downloaded and analyzed in a spreadsheet. The use of this list was possible only for positions in which one of the goat variant aminoacids matched the homologue in rCRS. The list includes the MutPred pathogenicity score, which is determined by a set of features reflecting protein structure and its dynamics, the presence of functional residues, biases of amino acid sequence, and evolutionary conservation at the substitution site and in its neighborhood. This score ranges from 0 (null pathogenicity) to 1 (maximum pathogenicity). Also included is the mtDNA selection score, defined as the ratio between the probability of observing a substitution of a given pathogenicity among all substitutions and the probability of observing it in three major clades of the human mtDNA tree. Values lower than 1 denote substitutions with likely weak or null detrimental effects.

Statistical tests were performed with R.

## Supporting Information

Figure S1 **Linearized map of goat mtDNA (at bottom).** The positions of the 33 amplicons used to sequence the entire molecule are shown in the lower panel as black segments. Note that Fragment 33 spans mtDNA position 1. A representation of the coverage of the entire mitogenome in 28 individuals is given in the top panel. Lines are colored according to D-loop clade affiliation (reported on the left, after individual id.). Grayed segments indicate portions not covered by sequencing or producing poor results. Individual id's are as in [12].
(JPG)

Figure S2 **Phylogenetic tree of 28 goat mtDNAs (positions 1-15,430) plus two reference sequences, obtained with BEAST.** Individual id's and D-loop clade affiliations are reported at the tips of the tree. The posterior for each node is shown next to it, in italics. The scale bar is in mutation units. The table at right shows the assignment of domestic goat sequences to each clade (based solely on D-loop sequences, according to criteria of ref. [12]) and their geographic provenance. Weu  =  western Europe (ref. [5]); Ceu  =  central Europe (ref. [5]); Meu  =  Mediterranean Europe excluding Sardinia (ref. [5]); Sar  =  Sardinia (refs. [12]+ [18]); Naf  =  north Africa (refs. [5]+ [13]); Ssaf  =  sub-saharan Africa (ref. [5]); Cas  =  central Asia (ref. [5]); Eas  =  eastern Asia (ref. [5]). Only one line is given for tree tips corresponding to clades A5 and A8. The corresponding assignments for the 29 haplogroup A bezoar (*C. a.)* sequences (ref. [6]) are also reported. 1. includes an ambiguous A7/A9 assignment; 2. includes an ambiguous A8/A9 assignment.
(JPG)

Figure S3 **Ratio between the number of non-synonymous and synonymous variants in the 13 mtDNA protein-coding genes among all variants recorded in MITOMAP (black bars), variants with >40 records in GenBank (grey bars) and variants found in this study (white bars).**
(JPG)

Figure S4 **Portions of the electropherograms of individuals 1237 and 1300 for amplicon 3, showing a double peak at pos. 4587.**
(PPTX)

Figure S5 **Same network as in** **Fig. 1** **with numbered branches.** The assignment of the mutations listed in Table S2 in File S1 to each branch follows this numbering system.
(JPG)

File S1 **Supporting tables and text.**
(DOCX)
