# Supplementary figures and images for: Phylogeny and Patterns of Diversity of Goat mtDNA Haplogroup A Revealed by Resequencing Complete Mitogenomes
Source: PLoS One. 2014 Apr 24;9(4):e95969. doi: 10.1371/journal.pone.0095969 (PMC3999278; doi:10.1371/journal.pone.0095969)

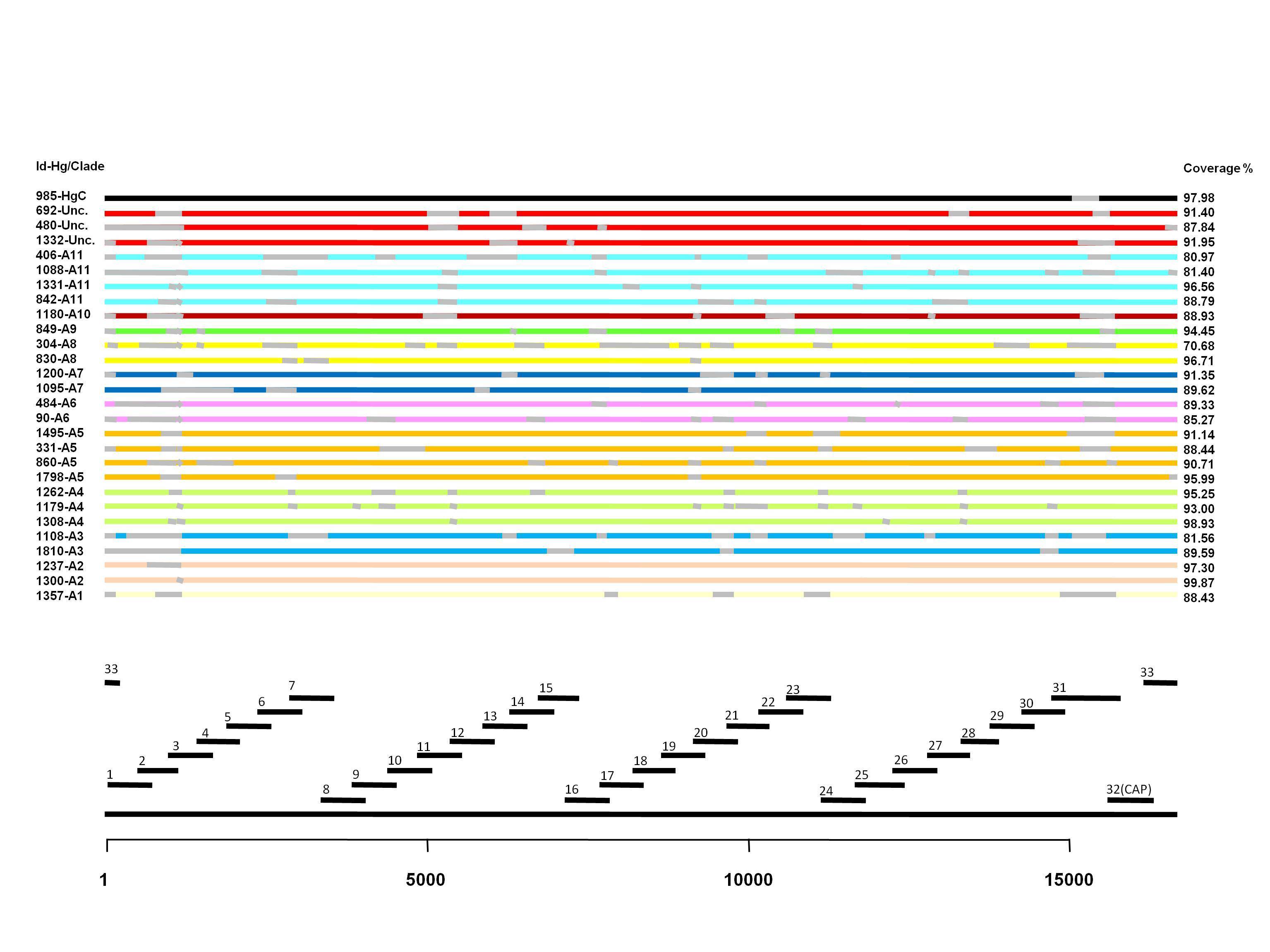

Supplement: Figure S1 — Linearized map of goat mtDNA (at bottom). The positions of the 33 amplicons used to sequence the entire molecule are shown in the lower panel as black segments. Note that Fragment 33 spans mtDNA position 1. A representation of the coverage of the entire mitogenome in 28 individuals is given in the top panel. Lines are colored according to D-loop clade affiliation (reported on the left, after individual id.). Grayed segments indicate portions not covered by sequencing or producing poor results. Individual id's are as in [12]. (JPG) [file pone.0095969.s001.jpg]

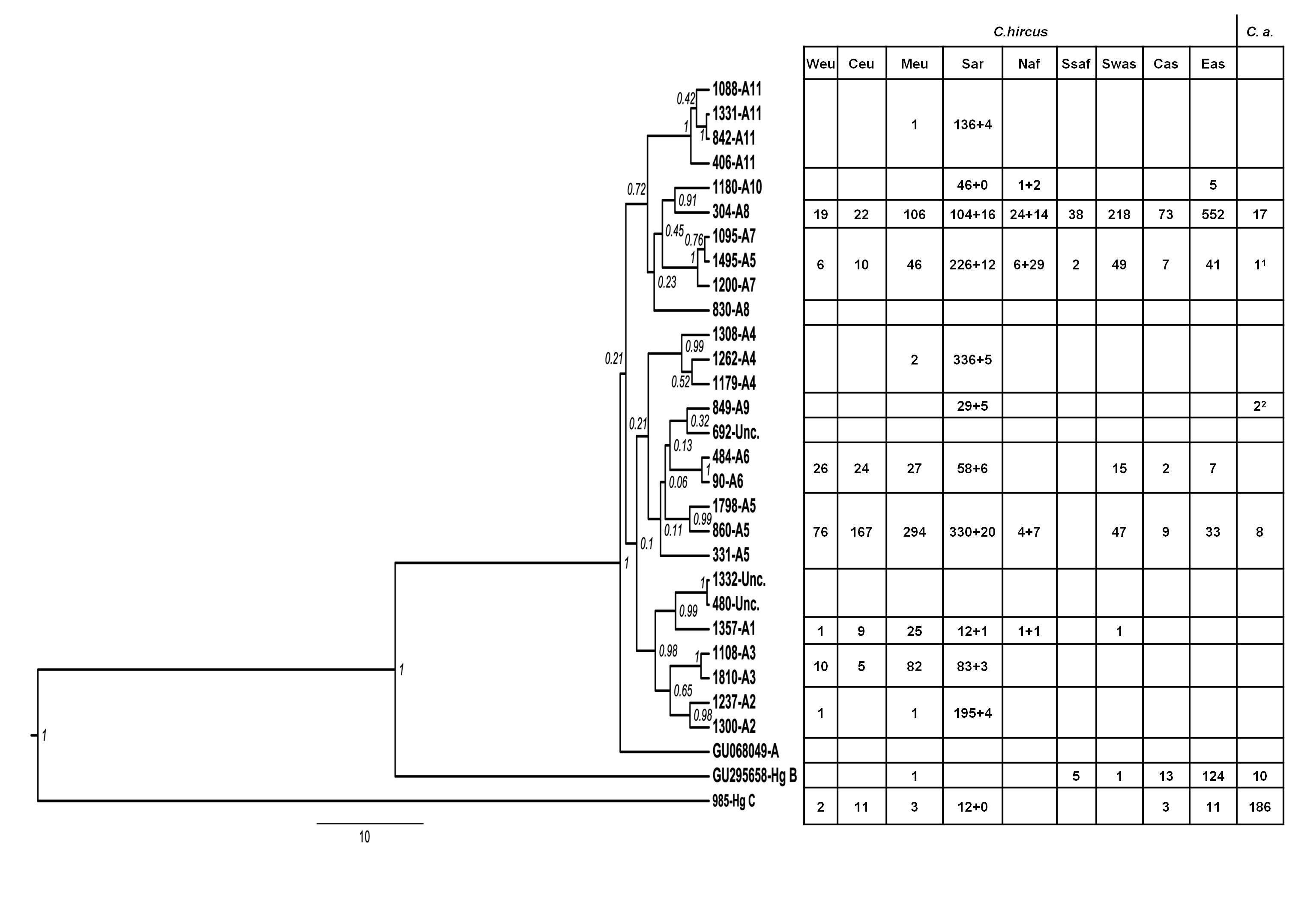

Supplement: Figure S2 — Phylogenetic tree of 28 goat mtDNAs (positions 1-15,430) plus two reference sequences, obtained with BEAST. Individual id's and D-loop clade affiliations are reported at the tips of the tree. The posterior for each node is shown next to it, in italics. The scale bar is in mutation units. The table at right shows the assignment of domestic goat sequences to each clade (based solely on D-loop sequences, according to criteria of ref. [12]) and their geographic provenance. Weu = western Europe (ref. [5]); Ceu = central Europe (ref. [5]); Meu = Mediterranean Europe excluding Sardinia (ref. [5]); Sar = Sardinia (refs. [12]+ [18]); Naf = north Africa (refs. [5]+ [13]); Ssaf = sub-saharan Africa (ref. [5]); Cas = central Asia (ref. [5]); Eas = eastern Asia (ref. [5]). Only one line is given for tree tips corresponding to clades A5 and A8. The corresponding assignments for the 29 haplogroup A bezoar (C. a.) sequences (ref. [6]) are also reported. 1. includes an ambiguous A7/A9 assignment; 2. includes an ambiguous A8/A9 assignment. (JPG) [file pone.0095969.s002.jpg]

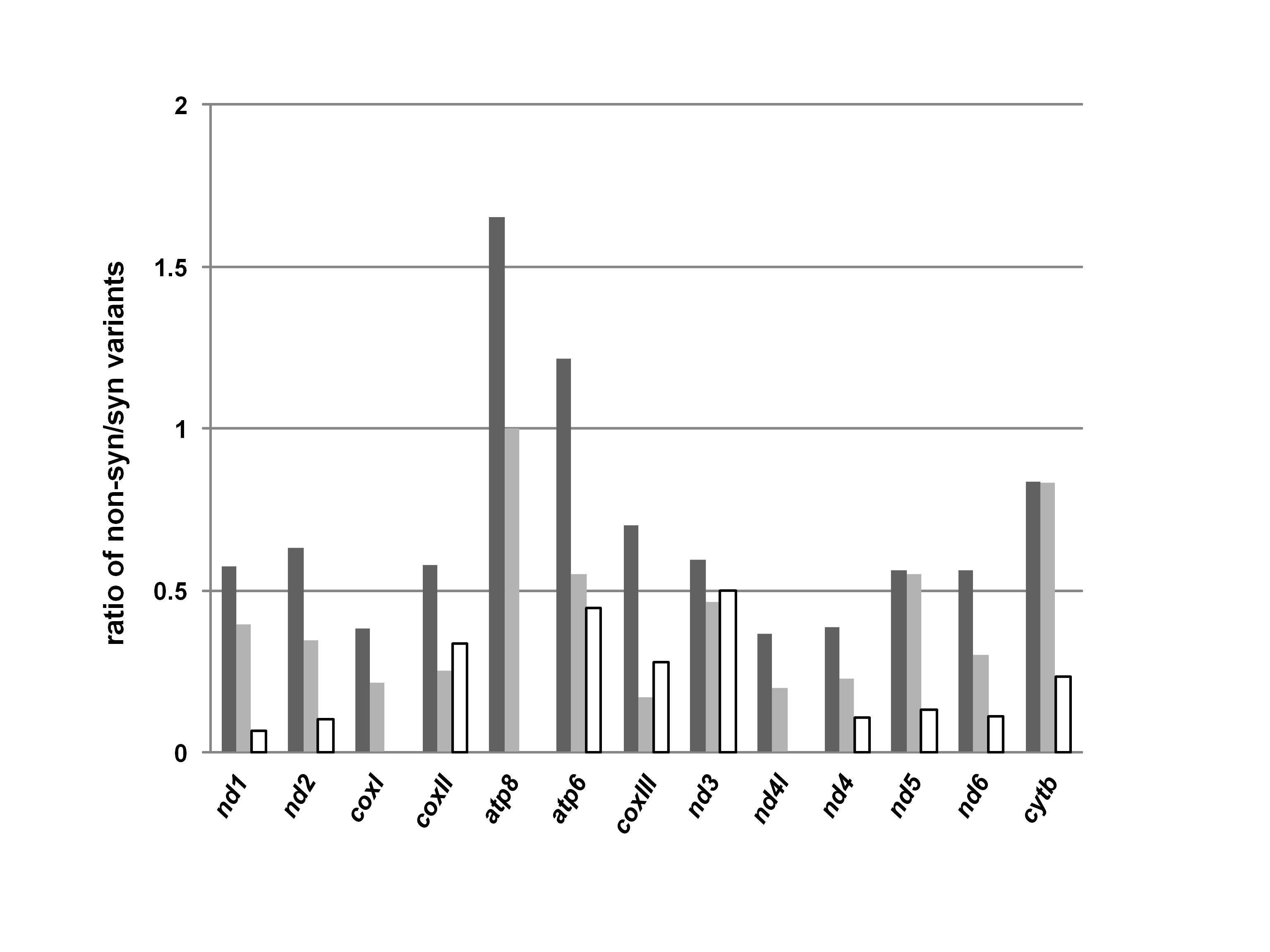

Supplement: Figure S3 — Ratio between the number of non-synonymous and synonymous variants in the 13 mtDNA protein-coding genes among all variants recorded in MITOMAP (black bars), variants with >40 records in GenBank (grey bars) and variants found in this study (white bars). (JPG) [file pone.0095969.s003.jpg]

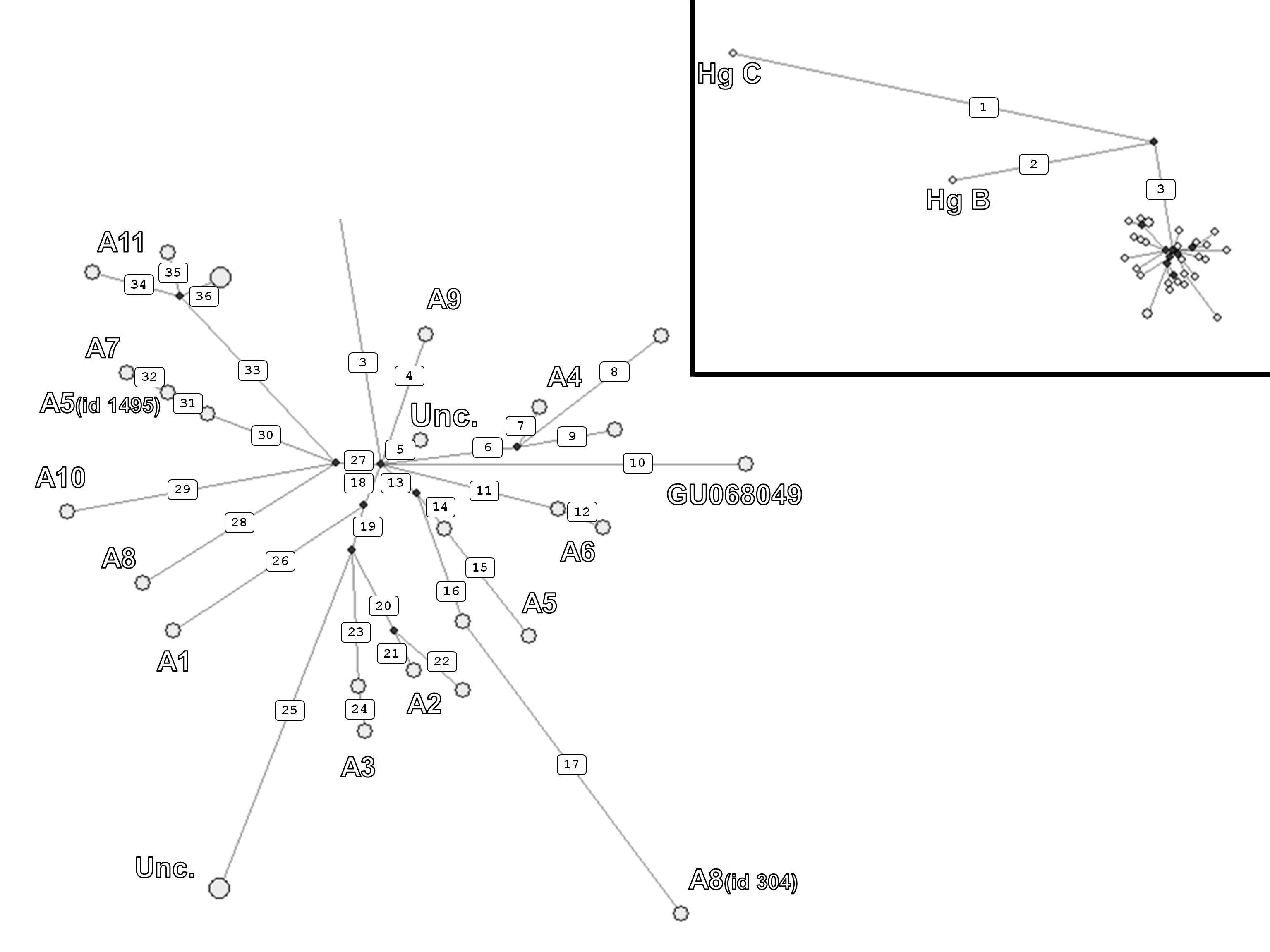

Supplement: Figure S5 — Same network as in Fig. 1 with numbered branches. The assignment of the mutations listed in Table S2 in File S1 to each branch follows this numbering system. (JPG) [file pone.0095969.s005.jpg]
