# Supplementary material for: Phylogeny and Patterns of Diversity of Goat mtDNA Haplogroup A Revealed by Resequencing Complete Mitogenomes
Source: PLoS One. 2014 Apr 24;9(4):e95969. doi: 10.1371/journal.pone.0095969 (PMC3999278; doi:10.1371/journal.pone.0095969)
Supplement: File S1 — Supporting tables and text. (DOCX) [file pone.0095969.s006.docx]

Supplemental text

Weaknesses and uncertainties in applying absolute genetic dating.

We explored the reliability of absolute genetic dates for the coalescent of Hg A and its internal nodes as obtained with BEAST under two distinct approaches: 1) using substitution rates obtained from the literature and 2) using a calibrated date for the tree root. In all cases run length, sampling and burn-in were as described in Materials and methods.

1a) We considered the average of the 8 rates reported in ref. [1] for the 3^rd^ codon positions in the genus *Capra* (4.5 E-8/site/year). This required the use of only 140 variant sites (Suppl. Table 2), which severely affected the tree topology, due to the exclusion of the rRNA, tRNA and 1^st^ and 2^nd^ codon positions, which comprehensively provide a relevant quota of phylogenetic information. Under these conditions the node basal to Hg A was estimated at 36.7 kya (95% HPD 23.6-51.4 kya);

1b) We also considered the rates reported for all protein-coding genes in several species or group of species (which do not include the goat) with Bayesian methods (Table 2 in ref. [2]). We used the average rate calculated both excluding (1.01 E-8/site/year) or including (1.27 E-8/site/year) *Mus musculus,* recognized by the same authors as outlier. This involved the use of only 171 variable positions (Suppl. Table 2). Under these conditions the node basal to Hg A was estimated at 61.0 (95% HPD 42.8-80.6 kya) and 48.5 kya (95% HPD 33.9-64.1 kya), respectively;

2) In this case we calibrated the rate in our tree by considering the midpoint of the dates proposed in ref. [3] for the coalescent of Hg’s A, B and C (240 kya), and in turn based on variation in *cytb* and the D-loop in the goat-sheep divergence. In this case we could use all variable positions, obtaining the same tree topology as in Suppl. Fig. 2. Under these conditions the node basal to Hg A was estimated at 30.7 kya (95% HPD 17.7-48.8 kya).

As each of this procedures relied on unwarranted assumptions and some degree of loss of information, we preferred to present our results in terms of mutational units.

Table S1. Id, provenance, breed affiliation, haplogroup and D-loop clade assignment of the 28 and 38 individuals selected for complete (above) and partial (below) mtDNA sequencing, respectively.

| Individual id^1^ | Sardinian sub-region | Breed | Main haplogroup | D-loop clade^1^ |
| --- | --- | --- | --- | --- |
|  |  |  |  |  |
| 1357 | GERREI | Sarda | A | A1 |
| 1300 | OGLIASTRA | Sarda | A | A2 |
| 1237 | OGLIASTRA | Sarda | A | A2 |
| 1810 | OGLIASTRA | Sarda | A | A3 |
| 1108 | OGLIASTRA | Sarda | A | A3 |
| 1308 | OGLIASTRA | Sarda | A | A4 |
| 1179 | OGLIASTRA | Sarda | A | A4 |
| 1262 | OGLIASTRA | Sarda | A | A4 |
| 1798 | OGLIASTRA | Sarda | A | A5 |
| 860 | OGLIASTRA | Sarda | A | A5 |
| 331 | SULCIS | Sarda | A | A5 |
| 1495 | SULCIS | Sarda | A | A5 |
| 90 | OGLIASTRA | Sarda | A | A6 |
| 484 | OGLIASTRA | Sarda | A | A6 |
| 1095 | OGLIASTRA | Sarda | A | A7 |
| 1200 | OGLIASTRA | Sarda | A | A7 |
| 830 | OGLIASTRA | Sarda | A | A8 |
| 304 | OGLIASTRA | Sarda | A | A8 |
| 849 | OGLIASTRA | Sarda | A | A9 |
| 1180 | OGLIASTRA | Sarda | A | A10 |
| 842 | OGLIASTRA | Sarda | A | A11 |
| 1331 | OGLIASTRA | Sarda | A | A11 |
| 1088 | OGLIASTRA | Sarda | A | A11 |
| 406 | OGLIASTRA | Sarda | A | A11 |
| 1332 | OGLIASTRA | Sarda | A | Unclassified |
| 480 | OGLIASTRA | Sarda | A | Unclassified |
| 692 | OGLIASTRA | Sarda | A | Unclassified |
| 985 | IGLESIENTE | Sarda | C | n.a. |
|  |  |  |  |  |
|  |  |  |  |  |
| 753 | GERREI | Sarda | A | A2 |
| 1911 | GERREI | Sarda | A | A2 |
| 318 | SULCIS | Sarda | A | A2 |
| 521 | SULCIS | Sarda | A | A2 |
| 906 | SARRABUS | Sarda | A | A2 |
| 879 | SARRABUS | Sarda | A | A2 |
| 554 | IGLESIENTE | Sarda | A | A2 |
| 1615 | IGLESIENTE | Sarda | A | A2 |
| 243 | GERREI | Sarda | A | A4 |
| 1015 | GERREI | Sarda | A | A4 |
| 1120 | SULCIS | Sarda | A | A4 |
| 905 | SULCIS | Sarda | A | A4 |
| 881 | SARRABUS | Sarda | A | A4 |
| 910 | SARRABUS | Sarda | A | A4 |
| 639 | IGLESIENTE | Sarda | A | A4 |
| 994 | IGLESIENTE | Sarda | A | A4 |
| 247 | GERREI | Sarda | A | A5 |
| 1019 | GERREI | Sarda | A | A5 |
| 1522 | SULCIS | Sarda | A | A5 |
| 178 | SULCIS | Sarda | A | A5 |
| 621 | SARRABUS | Sarda | A | A5 |
| 890 | SARRABUS | Sarda | A | A5 |
| 769 | IGLESIENTE | Sarda | A | A5 |
| 558 | IGLESIENTE | Sarda | A | A5 |
| 30 | GERREI | Sarda | A | A7 |
| 1781 | GERREI | Sarda | A | A7 |
| 1550 | SULCIS | Sarda | A | A7 |
| 909 | SARRABUS | Sarda | A | A7 |
| 1608 | IGLESIENTE | Sarda | A | A7 |
| 1607 | IGLESIENTE | Sarda | A | A7 |
| 1455 | SULCIS | Sarda | A | A8 |
| 1855 | SARRABUS | Sarda | A | A8 |
| 1771 | GERREI | Sarda | A | A11 |
| 1620 | GERREI | Sarda | A | A11 |
| 588 | SULCIS | Sarda | A | A11 |
| 216 | SULCIS | Sarda | A | A11 |
| 912 | SARRABUS | Sarda | A | A11 |
| 883 | SARRABUS | Sarda | A | A11 |

1. according to [4]

n.a. = not applicable

Table S2. List of variable positions in the sequences analyzed in this work

| Position | Gene | Allelic variants | Variable in Hg A (Y/N) | Network branch | Position in codon | Details | Amplicon |
| --- | --- | --- | --- | --- | --- | --- | --- |
| 165 | 12S-rRNA | T/C |  | 1 |  |  | 1 |
| 177 | 12S-rRNA | T/del | Y^2^ | 10^7^ |  |  | 1 |
| 178 | 12S-rRNA | T/del | Y^2^ | 10^7^ |  |  | 1 |
| 180 | 12S-rRNA | G/T | Y^2^ | 10 |  |  | 1 |
| 181 | 12S-rRNA | A/T | Y^2^ | 10 |  |  | 1 |
| 196 | 12S-rRNA | T/C | Y | 17,27 |  |  | 1 |
| 205 | 12S-rRNA | T/C |  | 2 |  |  | 1 |
| 213 | 12S-rRNA | T/C |  | 1 |  |  | 1 |
| 605 | 12S-rRNA | T/C | Y | 26^7^ |  |  | 2 |
| 944 | 12S-rRNA | T/C |  | 1^7^ |  |  | 2 |
| 1026 | 12S-rRNA | T/C |  | 1^7^ |  |  | 2 |
| 1114 | 16S-rRNA | T/C |  | 1 |  |  | 3 |
| 1111-1120 | 16S-rRNA | C_10_/C_11_ |  | 1^7^ |  |  | 3 |
| 1192 | 16S-rRNA | T/C |  | 1^7^ |  |  | 3 |
| 1238/39 | 16S-rRNA | A/del | Y | 32^7^ |  |  | 3 |
| 1369 | 16S-rRNA | T/C |  | 1^7^ |  |  | 3 |
| 1671 | 16S-rRNA | A/G | Y | 4 |  |  | 4 |
| 1850 | 16S-rRNA | T/C |  | 3 |  |  | 4 |
| 2120 | 16S-rRNA | T/C | Y | 28 |  |  | 5 |
| 2190 | 16S-rRNA | A/G | Y | 6 |  |  | 5 |
| 2208 | 16S-rRNA | T/C |  | 3 |  |  | 5 |
| 2619 | 16S-rRNA | T/C | Y | 26 |  |  | 6 |
| 2767 | *nd1* | T/C | Y | 26 | 1 | **F9L** | 6 |
| 2850 | *nd1* | T/C |  | 2 | 3 | G36G | 6 |
| 2886 | *nd1* | A/C |  | 1^7^ | 3 | P48P | 6 |
| 2971 | *nd1* | T/C | Y | 30 | 1 | L77L | 7 |
| 3003 | *nd1* | T/C |  | 1 | 3 | I87I | 7 |
| 3040 | *nd1* | T/C | Y | 4 | 1 | L100L | 7 |
| 3108 | *nd1* | T/C | Y | 17 | 3 | A122A | 7 |
| 3132 | *nd1* | T/C |  | 1 | 3 | I130I | 7 |
| 3195 | *nd1* | A/G |  | 3 | 3 | L151L | 7 |
| 3240 | *nd1* | T/C | Y | 8 | 3 | I166I | 7 |
| 3294 | *nd1* | A/G |  | 1 | 3 | M184M | 7 |
| 3345 | *nd1* | T/C | Y | 12 | 3 | T201T | 7 |
| 3396 | *nd1* | A/G | Y | 17 | 3 | G218G | 7 |
| 3469 | *nd1* | T/C |  | 3 | 1 | L243L | 7 |
| 3600 | *nd1* | A/G |  | 1 | 3 | M286M | 8 |
| 3634 | *nd1* | T/C | Y | 33 | 1 | L298L | 8 |
| 3771 | tRNA-Gln | T/C |  | 1 |  | Acceptor stem | 8 |
| 3892 | tRNA-Met | T/C | Y | 28 |  | T-domain loop | 8 |
| 4033 | *nd2* | T/C | Y | 2,28 | 3 | P42P | 9 |
| 4084 | *nd2* | T/C | Y | 29 | 3 | Y59Y | 9 |
| 4333 | *nd2* | T/C | Y | 18 | 3 | L142L | 9 |
| 4570 | *nd2* | T/C |  | 1 | 3 | A221A | 10 |
| 4587 | *nd2* | T/C | Uncertain in id 1237 and 1300 but T in 6 additional individuals | 20 | 2 | **T227I** | 10 |
| 4606 | *nd2* | A/G | Y | 14^7^ | 3 | T233T | 10 |
| 4627 | *nd2* | A/G |  | 1 | 3 | M240M | 10 |
| 4708 | *nd2* | T/C |  | 1 | 3 | I267I | 10 |
| 4714 | *nd2* | A/G |  | 1 | 3 | E269E | 10 |
| 4822 | *nd2* | T/C | Y^3^ |  | 3 | F305F | 10 |
| 4891 | *nd2* | T/C |  | 1 | 3 | T328T | 10 |
| 4942 | *nd2* | T/C | Y^2^ | 10 | 3 | I345I | 10 |
| 5602 | *coxI* | T/C |  | 3 | 3 | D91D | 12 |
| 5713 | *coxI* | A/G |  | 1 | 3 | V128V | 12 |
| 5725 | *coxI* | A/G | Y | 33 | 3 | L132L | 12 |
| 5731 | *coxI* | T/C | Y | 11 | 3 | G134G | 12 |
| 5776 | *coxI* | T/C |  | 1 | 3 | S149S | 12 |
| 6031 | *coxI* | A/G |  | 1 | 3 | L234L | 13 |
| 6094 | *coxI* | T/C |  | 1 | 3 | S255S | 13 |
| 6097 | *coxI* | T/C |  | 2 | 3 | H256H | 13 |
| 6109 | *coxI* | T/C | Y | 17 | 3 | Y260Y | 13 |
| 6112 | *coxI* | T/C | Y | 23 | 3 | Y261Y | 13 |
| 6187 | *coxI* | T/C | Y | 11 | 3 | I286I | 13 |
| 6325 | *coxI* | T/C |  | 1 | 3 | I332I | 13 |
| 6391 | *coxI* | T/C |  | 1 | 3 | T354T | 13 |
| 6415 | *coxI* | T/C |  | 1 | 3 | S362S | 13 |
| 6523 | *coxI* | T/C | Y^2^ | 10 | 3 | P398P | 13 |
| 6538 | *coxI* | T/C |  | 1 | 3 | Y403Y | 14 |
| 6700 | *coxI* | T/C | Y | 21 | 3 | G457G | 14 |
| 6894 | tRNA-Ser (UCN) | T/C | Y | 36 |  | T-domain stem | 14 |
| 7053 | *coxII* | A/G |  | 2 | 1 | **T13A** | 15 |
| 7118 | *coxII* | T/C | Y | 1,17,29 | 3 | I34I | 15 |
| 7185 | *coxII* | A/G | Y | 17 | 1 | **D57N** | 15 |
| 7214 | *coxII* | T/C | Y | 16,29,31 | 3 | T66T | 15 |
| 7343 | *coxII* | A/G |  | 2 | 3 | E109E | 16 |
| 7451 | *coxII* | T/C | Y | 26 | 3 | P145P | 16 |
| 7658 | *coxII* | T/C |  | 1 | 3 | V214V | 16 |
| 7661 | *coxII* | T/C |  | 2 | 3 | P215P | 16 |
| 7723 | tRNA-Lys | A/G | Y | 9 |  | Connector 2 | 16 |
| 7840 | *atp8* | T/C |  | 3 | 3 | I23I | 17 |
| 7886 | *atp8* | T/C |  | 2 | 1 | L39L | 17 |
| 7918 | *atp8* | T/C |  | 1 | 3 | P49P | 17 |
| 8056 | *atp6* | A/G |  | 3 | 1 | **V43I** | 17 |
| 8070 | *atp6* | A/G | Y | 34 | 3 | Q47Q | 17 |
| 8079 | *atp6* | T/C |  | 3 | 3 | L50L | 17 |
| 8114 | *atp6* | A/G |  | 1 | 2 | **N62S** | 17 |
| 8134 | *atp6* | A/G | Y | 25 | 1 | **T69A** | 17 |
| 8265 | *atp6* | T/C |  | 1 | 3 | A112A | 17 |
| 8301 | *atp6* | A/C |  | 1 | 3 | S124S | 18 |
| 8481 | *atp6* | T/C |  | 2 | 3 | I184I | 18 |
| 8487 | *atp6* | T/C |  | 1 | 3 | P186P | 18 |
| 8571 | *atp6* | T/C | Y | 29 | 3 | F214F | 18 |
| 8586 | *atp6* | T/C | Y | 15 | 3 | S219S | 18 |
| 8596 | *atp6* | T/C | Y | 16 | 1 | **H223Y** | 18 |
| 8604 | *atp6* | T/C | Y | 8 | 3 | N225N | 18 |
| 8636 | *coxIII* | T/C |  | 2 | 3 | H9H | 18 |
| 8704 | *coxIII* | T/C |  | 2 | 2 | **I32T** | 18 |
| 8731 | *coxIII* | T/C |  | 2 | 2 | **A41V** | 18 |
| 8735 | *coxIII* | A/G | Y | 25,26 | 3 | L42L | 18 |
| 8759 | *coxIII* | T/C | Y | 33 | 3 | N50N | 18 |
| 8819 | *coxIII* | T/C |  | 2 | 3 | H70H | 19 |
| 8852 | *coxIII* | T/C |  | 1 | 3 | Y81Y | 19 |
| 8915 | *coxIII* | T/C | Y | 11 | 3 | Y102Y | 19 |
| 8939 | *coxIII* | T/C | Y | 22 | 3 | P110P | 19 |
| 8960 | *coxIII* | T/C |  | 3 | 3 | P117P | 19 |
| 9011 | *coxIII* | T/C |  | 2 | 3 | T134T | 19 |
| 9015 | *coxIII* | A/G | Y | 25 | 1 | **V136I** | 19 |
| 9062 | *coxIII* | T/C |  | 1 | 3 | L151L | 19 |
| 9065 | *coxIII* | A/G | Y | 16 | 3 | M152M | 19 |
| 9083 | *coxIII* | T/C | Y | 22 | 3 | H158H | 19 |
| 9112 | *coxIII* | T/C |  | 1 | 2 | **M168T** | 19 |
| 9206 | *coxIII* | A/G |  | 1^7^ | 3 | V199V | 19 |
| 9221 | *coxIII* | T/C | Y | 11 | 3 | H204H | 19 |
| 9224 | *coxIII* | T/C | Y | 29 | 3 | G205G | 19 |
| 9257 | *coxIII* | T/C | Y | 8 | 3 | I216I | 20 |
| 9308 | *coxIII* | T/C | Y | 28 | 3 | F233F | 20 |
| 9347 | *coxIII* | T/C |  | 1 | 3 | D246D | 20 |
| 9369 | *coxIII* | C/G | Y | 34 | 1 | **V254L** | 20 |
| 9548 | *nd3* | T/C |  | 1 | 2 | **V29A** | 20 |
| 9600 | *nd3* | A/G |  | 1^7^ | 3 | S46S | 20 |
| 9738 | *nd3* | T/C | Y | 30 | 3 | L92L | 20 |
| 9827 | tRNA-Arg | T/C |  | 3 |  | D-domain loop | 21 |
| 9860 | tRNA-Arg | T/C | Y | 6 |  | T-domain loop | 21 |
| 9863 | tRNA-Arg | A/G | Y | 15 |  | T-domain loop | 21 |
| 9995 | *nd4l* | T/C |  | 1 | 3 | S39S | 21 |
| 10028 | *nd4l* | T/C | Y^2^ | 10 | 3 | N50N | 21 |
| 10107 | *nd4l* | T/C |  | 1 | 1 | L77L | 21 |
| 10145 | *nd4l* | T/C | Y | 23 | 3 | Y89Y | 21 |
| 10303 | *nd4* | T/C |  | 1 | 3 | F45F | 22 |
| 10357 | *nd4* | A/T |  | 1 | 3 | T63T | 22 |
| 10391 | *nd4* | T/C | Y | 25 | 1 | L75L | 22 |
| 10433 | *nd4* | T/C | Y | 33 | 1 | **P89S** | 22 |
| 10450 | *nd4* | T/C |  | 1 | 3 | L94L | 22 |
| 10480 | *nd4* | A/G |  | 2 | 3 | L104L | 22 |
| 10493 | *nd4* | A/G | Y | 24 | 1 | **T109A** | 22 |
| 10516 | *nd4* | T/C | Y^4^ |  | 3 | I116I | 22 |
| 10546 | *nd4* | A/G |  | 1 | 3 | L126L | 22 |
| 10564 | *nd4* | T/C |  | 1 | 3 | I132I | 22 |
| 10639 | *nd4* | T/C |  | 3 | 3 | S157S | 22 |
| 10687 | *nd4* | T/C |  | 2 | 3 | S173S | 22 |
| 10814 | *nd4* | T/C |  | 2 | 1 | L216L | 23 |
| 10945 | *nd4* | T/C | Y | 19 | 3 | Y259Y | 23 |
| 10951 | *nd4* | T/C |  | 1 | 3 | F261F | 23 |
| 11062 | *nd4* | T/C | Y^2^ | 10 | 3 | I298I | 23 |
| 11074 | *nd4* | T/C |  | 1 | 3 | L302L | 23 |
| 11128 | *nd4* | T/C | Y^2^ | 10^7^ | 3 | G320G | 23 |
| 11257 | *nd4* | T/C |  | 1 | 3 | S363S | 24 |
| 11258 | *nd4* | T/C | Y | 28 | 1 | L364L | 24 |
| 11365 | *nd4* | T/C |  | 2 | 3 | N399N | 24 |
| 11374 | *nd4* | T/C | Y | 14 | 3 | I402I | 24 |
| 11560-7 | tRNA-His | A_8_/A_9_ | Y | 2,26^7^ |  | D-domain loop-stem | 24 |
| 11569 | tRNA-His | A/G |  | 1 |  | Connector 2 | 24 |
| 11624 | tRNA-Ser (AGY) | T/C | Y | 25 |  | Connector 1 | 24 |
| 11691 | tRNA-Leu(CUN) | A/G |  | 2 |  | D-domain loop | 24 |
| 11702 | tRNA-Leu(CUN) | A/G |  | 2 |  | Connector 2 | 24 |
| 11872 | *nd5* | A/G |  | 2 | 2 | **C42Y** | 25 |
| 12146 | *nd5* | T/C |  | 1 | 3 | T133T | 25 |
| 12158 | *nd5* | T/C | Y | 8 | 3 | L137L | 25 |
| 12314 | *nd5* | T/C |  | 1 | 3 | F189F | 25 |
| 12362 | *nd5* | A/G | Y | 1,32 | 3 | K205K | 26 |
| 12380 | *nd5* | A/G | Y^2^ | 10 | 3 | L211L | 26 |
| 12383 | *nd5* | T/C |  | 3 | 3 | P212P | 26 |
| 12386 | *nd5* | A/G |  | 1 | 3 | L213L | 26 |
| 12404 | *nd5* | T/C | Y | 15 | 3 | A219A | 26 |
| 12539 | *nd5* | T/C |  | 1 | 3 | H264H | 26 |
| 12566 | *nd5* | T/C | Y | 25 | 3 | A273A | 26 |
| 12626 | *nd5* | T/C |  | 1 | 3 | L293L | 26 |
| 12638 | *nd5* | T/C |  | 1 | 3 | D297D | 26 |
| 12659 | *nd5* | T/C | Y | 20 | 3 | F304F | 26 |
| 12764 | *nd5* | A/G | Y | 9 | 3 | L339L | 26 |
| 12791 | *nd5* | T/C |  | 1 | 3 | H348H | 26 |
| 13001 | *nd5* | T/C |  | 1 | 3 | F418F | 27 |
| 13034 | *nd5* | T/C | Y^2^ | 10 | 3 | F429F | 27 |
| 13100 | *nd5* | A/T |  | 2 | 3 | **I451M** | 27 |
| 13157 | *nd5* | T/C |  | 1 | 3 | N470N | 27 |
| 13166 | *nd5* | T/C | Y | 4 | 3 | P473P | 27 |
| 13178 | *nd5* | T/C |  | 2 | 3 | I477I | 27 |
| 13280 | *nd5* | A/G |  | 2 | 3 | L511L | 27 |
| 13286 | *nd5* | T/C |  | 2 | 3 | F513F | 27 |
| 13292 | *nd5* | T/C |  | 1 | 3 | Y515Y | 27 |
| 13390 | *nd5* | T/C | Y | 29 | 2 | **S548L** | 28 |
| 13504 | *nd5* | A/T | Y^5^ |  | 2 | **L586Q** | 28 |
| 13663 | *nd6* | T/C | Y | 17 | 3 | E139E | 28 |
| 13681 | *nd6* | A/G |  | 2 | 3 | S133S | 28 |
| 13714 | *nd6* | T/C | Y | 14 | 3 | M122M | 28 |
| 13741 | *nd6* | T/C |  | 3 | 3 | V113V | 28 |
| 13756 | *nd6* | T/C |  | 3 | 3 | L108L | 28 |
| 13762 | *nd6* | A/G |  | 2 | 3 | Y106Y | 28 |
| 13945 | *nd6* | T/C | Y | 5 | 3 | L45L | 29 |
| 13964 | *nd6* | A/G |  | 2 | 2 | **V39A** | 29 |
| 14044 | *nd6* | A/G |  | 1 | 3 | I12I | 29 |
| 14053 | *nd6* | T/C | Y | 35 | 3 | L9L | 29 |
| 14290 | *cytb* | A/G |  | 1 | 3 | L46L | 29 |
| 14386 | *cytb* | T/C |  | 1 | 3 | I78I | 30 |
| 14461 | *cytb* | T/C |  | 2 | 3 | Y103Y | 30 |
| 14479 | *cytb* | A/T | Y | 23 | 3 | **F109L** | 30 |
| 14497 | *cytb* | T/C | Y | 33 | 3 | I115I | 30 |
| 14518 | *cytb* | A/G |  | 1 | 3 | A122A | 30 |
| 14527 | *cytb* | T/C |  | 1 | 3 | A125A | 30 |
| 14617 | *cytb* | T/C | Y | 25,26 | 3 | Y155Y | 30 |
| 14746 | *cytb* | T/C |  | 3 | 3 | L198L | 30 |
| 14795 | *cytb* | A/G |  | 2 | 1 | **T215A** | 30 |
| 14865 | *cytb* | T/C | Y | 30 | 2 | **V238A** | 30 |
| 14917 | *cytb* | T/C | Y | 25 | 3 | N255N | 31 |
| 15004 | *cytb* | T/C |  | 3 | 3 | I284I | 31 |
| 15053 | *cytb* | T/C |  | 1 | 1 | L301L | 31 |
| 15055 | *cytb* | A/G | Y | 7 | 3 | L301L | 31 |
| 15220 | *cytb* | T/C |  | 2^7^ | 3 | I356I | 31 |
| 15295 |  | A/G | Y | *tip of* 25^6^ |  |  | 31 |

1. tRNA details determined by comparison with [5];for aminoacid coding positions the aminoacid represented in GU068049 is listed first; non-synonymous changes are bolded.
2. variant only in GU068049;
3. variant only in two additional individuals, both carriers of D-loop clade A5;
4. variant only in one additional individual, carrier of D-loop clade A5;
5. variant only in one additional individual, carrier of D-loop clade A11;
6. this mutation differentiates two sequences (id. 480 and 1332) that appear lumped in the network;
7. mutation not considered in network construction (see Materials and methods) and assigned *a posteriori* upon visual inspection.

Table S3. Counts of non-synonymous and synonymous variants found among the 30 sequences of the full set (Hg’s A, B and C), within Hg A only, and in the divergence with *C. ibex*.

|  |  | Non-synonymous | Synonymous | Fisher's Exact Test  (in comparison with divergence) | Proportion of  non-synonymous |
| --- | --- | --- | --- | --- | --- |
|  |  |  |  |  |  |
| *nd1* |  |  |  |  |  |
|  | Polymorphic in Hg's A,B,C | 1 | 15 | p-value = 1 | 0.063 |
|  | Polymorphic in Hg A | 1 | 7 | p-value = 1 | 0.125 |
|  | Divergence | 4 | 39 |  | 0.093 |
|  |  |  |  |  |  |
| *nd2* |  |  |  |  |  |
|  | Polymorphic in Hg's A,B,C | 1 | 10 | p-value = 0.6809 | 0.091 |
|  | Polymorphic in Hg A | 1 | 5 | p-value = 1 | 0.167 |
|  | Divergence | 14 | 62 |  | 0.184 |
|  |  |  |  |  |  |
| *coI* |  |  |  |  |  |
|  | Polymorphic in Hg's A,B,C | 0 | 17 | p-value = 1 | 0.000 |
|  | Polymorphic in Hg A | 0 | 7 | p-value = 1 | 0.000 |
|  | Divergence | 3 | 82 |  | 0.035 |
|  |  |  |  |  |  |
| *coII* |  |  |  |  |  |
|  | Polymorphic in Hg's A,B,C | 2 | 6 | p-value = 0.0708 | 0.250 |
|  | Polymorphic in Hg A | 1 | 3 | p-value = 0.1794 | 0.250 |
|  | Divergence | 1 | 38 |  | 0.026 |
|  |  |  |  |  |  |
| *atp8* |  |  |  |  |  |
|  | Polymorphic in Hg's A,B,C | 0 | 3 | p-value = 0.2 | 0.000 |
|  | Polymorphic in Hg A | 0 | 0 | n.a. |  |
|  | Divergence | 7 | 5 |  | 0.583 |
|  |  |  |  |  |  |
| *atp6* |  |  |  |  |  |
|  | Polymorphic in Hg's A,B,C | 4 | 9 | p-value = 0.7161 | 0.308 |
|  | Polymorphic in Hg A | 2 | 4 | p-value = 0.6302 | 0.333 |
|  | Divergence | 9 | 29 |  | 0.237 |
|  |  |  |  |  |  |
| *coIII* |  |  |  |  |  |
|  | Polymorphic in Hg's A,B,C | 5 | 18 | p-value = 0.3061 | 0.217 |
|  | Polymorphic in Hg A | 2 | 10 | p-value = 0.6455 | 0.167 |
|  | Divergence | 6 | 44 |  | 0.120 |
|  |  |  |  |  |  |
| *nd3* |  |  |  |  |  |
|  | Polymorphic in Hg's A,B,C | 1 | 2 | p-value = 0.3080 | 0.333 |
|  | Polymorphic in Hg A | 0 | 1 | p-value = 1 | 0.000 |
|  | Divergence | 2 | 22 |  | 0.083 |
|  |  |  |  |  |  |
| *nd4l* |  |  |  |  |  |
|  | Polymorphic in Hg's A,B,C | 0 | 4 | p-value = 1 | 0.000 |
|  | Polymorphic in Hg A | 0 | 2 | p-value = 1 | 0.000 |
|  | Divergence | 1 | 12 |  | 0.077 |
|  |  |  |  |  |  |
| *nd4* |  |  |  |  |  |
|  | Polymorphic in Hg's A,B,C | 2 | 19 | p-value = 0.735 | 0.095 |
|  | Polymorphic in Hg A | 2 | 6 | p-value = 0.3420 | 0.250 |
|  | Divergence | 14 | 85 |  | 0.141 |
|  |  |  |  |  |  |
| *nd5* |  |  |  |  |  |
|  | Polymorphic in Hg's A,B,C | 3 | 23 | p-value = 0.5732 | 0.115 |
|  | Polymorphic in Hg A | 1 | 9 | p-value = 1 | 0.100 |
|  | Divergence | 25 | 112 |  | 0.182 |
|  |  |  |  |  |  |
| *nd6* |  |  |  |  |  |
|  | Polymorphic in Hg's A,B,C | 1 | 9 | p-value = 1 | 0.100 |
|  | Polymorphic in Hg A | 0 | 4 | p-value = 1 | 0.000 |
|  | Divergence | 5 | 26 |  | 0.161 |
|  |  |  |  |  |  |
| *cytb* |  |  |  |  |  |
|  | Polymorphic in Hg's A,B,C | 3 | 13 | p-value = 1 | 0.188 |
|  | Polymorphic in Hg A | 2 | 4 | p-value = 0.3130 | 0.333 |
|  | Divergence | 16 | 74 |  | 0.178 |
|  |  |  |  |  |  |
| *Total* |  |  |  |  |  |
|  | Polymorphic in Hg's A,B,C | 23 | 148 | p-value = 0.8088 | 0.135 |
|  | Polymorphic in Hg A | 12 | 62 | p-value = 0.7299 | 0.162 |
|  | Divergence | 107 | 630 |  | 0.145 |

Table S4. Positions and primers for the 33 amplicons used in this work

| Amplicon | Primer names | Genomic positions^1^ | Primer sequences  (5’->3’) | Annealing  temp. (°C) |
| --- | --- | --- | --- | --- |
|  |  |  |  |  |
| 1 | 1F | 43-62 | AGATGAGTGTACCAACTCCA | 55° |
|  | 1R | 644-663 | AGGATTGGTGAGGTTTATCG |  |
| 2 | 2F | 506-525 | CTATGCTTAGCCCTAAACAC | 54° |
|  | 2R | 1046-1065 | CTAGGTGTAAACTAGGTGCT |  |
| 3 | 3F | 994-1013 | GGTAAGCATACTGGAAAGTG | 54° |
|  | 3R | 1590-1609 | TTAGGCCTACTATGGTGTTG |  |
| 4 | 4F | 1439-1458 | TGGTGATAGCTGGTTGTCCA | 58° |
|  | 4R | 2013-2031 | GCTACCTTTGCACGGTCAG |  |
| 5 | 5F | 1899-1918 | AAAGGAACTCGGCAAACACA | 58° |
|  | 5R | 2499-2517 | GCTCCGGTCTGAACTCAGA |  |
| 6 | 6F | 2372-2391 | ACAGCGCAATCCTATTCAAG | 56° |
|  | 6R | 2972-2991 | TAAGGCTAGGGTCAGAGCTA |  |
| 7 | 7F | 2877-2896 | ACTTCAACCAATCGCTGATG | 56° |
|  | 7R | 3474-3493 | TATAGGGGCTGTGAAATGCT |  |
| 8 | 8F | 3363-3382 | AGTATCAGGCTTCAACGTAG | 54° |
|  | 8R | 3963-3982 | GTGGGAGCTAATTATGACGA |  |
| 9 | 9F | 3852-3871 | AATTAAGCTATCGGGCCCAT | 55° |
|  | 9R | 4452-4471 | ACCTATGTGGGCAATTGATG |  |
| 10 | 10F | 4411-4430 | AGGACTAAACCAAACCCAAC | 55° |
|  | 10R | 5011-5030 | CTTGCAGTCCTTATCAGGAA |  |
| 11 | 11F | 4871-4890 | CGAATAACTCTCCTACCAAC | 53° |
|  | 11R | 5471-5490 | TAGATCTGGTCATCTCCAAG |  |
| 12 | 12F | 5377-5396 | CACCCTCTACCTTCTGTTCG | 57° |
|  | 12R | 5976-5995 | TGGGTCAAAGAAGGTTGTGT |  |
| 13 | 13F | 5897-5916 | ATTACTGCCGTACTACTCCT | 55° |
|  | 13R | 6491-6510 | ACAAATCCCCCTATGATAGC |  |
| 14 | 14F | 6312-6331 | ACGGAGGCAATATCAAATGG | 56° |
|  | 14R | 6904-6923 | TATGGTGTTGGCTTGAAACC |  |
| 15 | 15F | 6749-6768 | GAAGCATTTGCATCCAAACG | 57° |
|  | 15R | 7289-7308 | TTACTGTGAGGGATGGGTTG |  |
| 16 | 16F | 7181-7200 | CATAGACGCACAAGAAGTAG | 54° |
|  | 16R | 7778-7797 | CATGTCGATGTGTCTAGTTG |  |
| 17 | 17F | 7710-7729 | GAAGCTATGTAGCGTTAACC | 56° |
|  | 17R | 8307-8326 | TTCCTTGTGGTAGGAAATGG |  |
| 18 | 18F | 8230-8249 | AATCTAGGCATGGCTATTCC | 54° |
|  | 18R | 8797-8816 | ACCTTGGAAGGTACTTTCTC |  |
| 19 | 19F | 8677-8696 | CCGCTCTCCTACTAACATCC | 57° |
|  | 19R | 9258-9277 | AGTTGACGGAAAAAGCAGAC |  |
| 20 | 20F | 9170-9189 | AATTTCAGACGGAGTCTACG | 55° |
|  | 20R | 9765-9784 | GAGTTCATTCGTAGGCTAGG |  |
| 21 | 21F | 9688-9707 | CCATGAGCCTCACAAACAAC | 57° |
|  | 21R | 10272-10291 | AAGGAGTAGGCTTGTGAAGC |  |
| 22 | 22F | 10198-10217 | ACTTATACCCCTAACCTGAC | 53° |
|  | 22R | 10790-10809 | AGGTGGAGTCCATATAATGG |  |
| 23 | 23F | 10630-10649 | AACAGGATCCCTACCCCTAC | 58° |
|  | 23R | 11226-11245 | TCATCAGGCAGCCATTAGTG |  |
| 24 | 24F | 11165-11184 | AACTATGAGCGAATCCACAG | 56° |
|  | 24R | 11761-11780 | GGTGGTTAGTGCAAGAGAAG |  |
| 25 | 25F | 11694-11713 | AGTTATCCGTTGGTCTTAGG | 54° |
|  | 25R | 12370-12389 | TATCAGGGGTAGGTTTGAGT |  |
| 26 | 26F | 12278-12297 | TGGAGACATTGGGTTTATCC | 54° |
|  | 26R | 12878-12897 | GTATTCCTGTTAGTGCAAGG |  |
| 27 | 27F | 12822-12841 | ATGGGAGGCCTATTTAAAGC | 55° |
|  | 27R | 13392-13411 | AGATCTAGGAGAGAGGATGC |  |
| 28 | 28F | 13339-13358 | CAATCATACACCGCTTAACC | 54° |
|  | 28R | 13838-13857 | GCTACTGAGCAATATCCTGA |  |
| 29 | 29F | 13793-13812 | CCAGTAACAAACGCCCCTAG | 58° |
|  | 29R | 14390-14409 | CCGTTTGCGTGTATGTATCG |  |
| 30 | 30F | 14284-14303 | AATCCTGACAGGCCTATTCC | 57° |
|  | 30R | 14879-14898 | AGGTCGGGTGTGAATAGTAC |  |
| 31 | 31F | 14747-14766 | TTCCTCCACGAAACAGGATC | 57° |
|  | 31R | 15746-15765 | TGCTGGATTAGTACTGCACA |  |
| CAP^2^ | CAP F | 15635-15654 | CGTGTATGCAAGTACATTAC | 56° |
|  | CAP R | 16253-16273 | CTGATTAGTCATTAGTCCATC |  |
| 32 | 32F | 16192-16211 | TCTTTCTTCAGGGCCATCTC | 57° |
|  | 32R | 138-157 | TGATTTGGAGGGCGTTACTC |  |

1. numbered as in the reference sequence GU068049;
2. same as in ref.[3]

REFERENCES FOR SUPPLEMENTAL MATERIALS

1. Nabholz B, Glèmin S, Galtier N (2008) Strong variations of mitochondrial mutation rate across mammals-the longevity hypothesis. Mol Biol Evol 25: 120-130.

2. Soares P, Abrantes D, Rito T, Thomson N, Radivojac P, et al. (2013) Evaluating purifying selection in the mitochondrial DNA of various mammalian species. PLoS ONE 8: e58993.

3. Luikart G, Gielly L, Excoffier L, Vigne J-D, Bouvet J, et al. (2001) Multiple maternal origins and weak phylogeographic structure in domestic goats. Proc Natl Acad Sci USA 98: 5927-5932.

4. Piras D, Doro MG, Casu G, Melis MP, Vaccargiu S, et al. (2012) Haplotype affinities resolve a major component of goat (*Capra hircus*) mtDNA D-loop diversity and reveal specific features of the Sardinian stock. PLoSONE 7: e30785.

5. Helm M, Brulé H, Friede D, Giegé R, Pütz D, et al. (2000) Search for characteristic structural features of mammalian mitochondrial tRNAs. RNA 6: 1356-1379.
